# *Pantoea ananatis* carotenoid production confers toxoflavin tolerance and is regulated by Hfq‐controlled quorum sensing

**DOI:** 10.1002/mbo3.1143

**Published:** 2020-12-02

**Authors:** Okhee Choi, Byeongsam Kang, Yongsang Lee, Yeyeong Lee, Jinwoo Kim

**Affiliations:** ^1^ Institute of Agriculture and Life Science Gyeongsang National University Jinju Korea; ^2^ Division of Applied Life Science Gyeongsang National University Jinju Korea; ^3^ Department of Plant Medicine Gyeongsang National University Jinju Korea

**Keywords:** carotenoid, ClpP, Hfq, *Pantoea ananatis*, quorum sensing, RpoS

## Abstract

Carotenoids are widely used in functional foods, cosmetics, and health supplements, and their importance and scope of use are continuously expanding. Here, we characterized carotenoid biosynthetic genes of the plant‐pathogenic bacterium *Pantoea ananatis*, which carries a carotenoid biosynthetic gene cluster (including *crtE*, *X*, *Y*, *I*, *B*, and *Z*) on a plasmid. Reverse transcription–polymerase chain reaction (RT‐PCR) analysis revealed that the *crtEXYIB* gene cluster is transcribed as a single transcript and *crtZ* is independently transcribed in the opposite direction. Using splicing by overlap extension with polymerase chain reaction (SOE by PCR) based on asymmetric amplification, we reassembled *crtE*–*B*, *crtE*–*B*–*I*, and *crtE*–*B*–*I*–*Y*. High‐performance liquid chromatography confirmed that *Escherichia coli* expressing the reassembled *crtE*–*B*, *crtE*–*B*–*I*, and *crtE*–*B*–*I*–*Y* operons produced phytoene, lycopene, and β‐carotene, respectively. We found that the carotenoids conferred tolerance to UV radiation and toxoflavin. *Pantoea ananatis* shares rice environments with the toxoflavin producer *Burkholderia glumae* and is considered to be the first reported example of producing and using carotenoids to withstand toxoflavin. We confirmed that carotenoid production by *P. ananatis* depends on RpoS, which is positively regulated by Hfq/ArcZ and negatively regulated by ClpP, similar to an important regulatory network of *E. coli* (Hfq^ArcZ^ →RpoS Ͱ ClpXP). We also demonstrated that Hfq‐controlled quorum signaling de‐represses EanR to activate RpoS, thereby initiating carotenoid production. Survival genes such as those responsible for the production of carotenoids of the plant‐pathogenic *P. ananatis* must be expressed promptly to overcome stressful environments and compete with other microorganisms. This mechanism is likely maintained by a brake with excellent performance, such as EanR.

## Graphical abstract

Carotenoid production confers tolerance to toxoflavin and UV radiation in *Pantoea ananatis*. We proposed a model of carotenoid production for the previously reported regulatory network Hfq^ArcZ^ → RpoS Ͱ ClpXP and that identified here, in which Hfq‐controlled quorum signaling derepresses EanR to activate RpoS expression, thereby initiating carotenoid production.

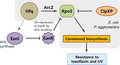

## 1 INTRODUCTION

Carotenoids are widely used in functional foods, cosmetics, and health supplements, and their importance and scope of use are continuously expanding (Ram et al., 2020; Song et al., 2013). Carotenoids are produced by plants and microorganisms, including algae, fungi, yeast, and bacteria, but animals must obtain carotenoids from dietary sources. Interestingly, aphids, which are capable of synthesizing carotenoids, are reported by later gene transfer from fungi (Moran & Jarvik, 2010). Several carotenoid‐producing bacteria have been identified (Dufossé et al., 2005; Fasano et al., 2014; Fidan & Zhan, 2019; Lorquin et al., 1997; Lu et al., 2017; Ram et al., 2020; Sajilata et al., 2008; Sedkova et al., 2005; Virtamo et al., 2014). Carotenoids are highly hydrophobic, restricted to essential parts of the complex membrane and cell wall in bacteria, and mainly responsible for enhancing various functions related to the cell membrane and walls, including physical strength, fluidity, cell wall rigidity, and lipid peroxidation (Kirti et al., 2014; Lutnaes et al., 2004; Vila et al., 2019). Several functions are closely related to the habitats of bacteria; in particular, the carotenoids of bacterial species living in low‐ or high‐temperature environments are used to control the membrane fluid, while those of bacteria continuously exposed to UV radiation increase tolerance to UV (Dundas & Larsen, 1963; Kunisawa & Stanier, 1958; Mathews & Sistrom, 1959, 1960; Mostofian et al., 2020; Stanier, 1959). Also, carotenoids aid bacteria in combating stresses related to oxidation, salt, and desiccation (Oren, 2009; Tian & Hua, 2010). When bacteria are placed in a stressful environment, carotenoid production increases to protect against particular stressors, such as temperature, salt, light, and acidity (Paliwal et al., 2017; Ram et al., 2019). This is consistent with the fact that bacterial carotenoid production is closely related to habitat characteristics.

Bacteria are surprisingly rich producers of carotenoids. However, bacteria with low carotenoid content are unsuitable for commercial use. The production of plant‐based carotenoids in bacteria is easier than in eukaryotic organisms such as yeasts, fungi, and plants (Ram et al., 2020). Previously, the biosynthesis of carotenoids has relied on bacterial carotenoid genes and DNA recombination techniques. Because these methods depend on restriction sites, generating recombinant DNA fragments and rearranging multiple carotenoid genes is problematic. The technique of splicing by overlap extension by polymerase chain reaction (SOE by PCR) using asymmetric amplification was first developed for introducing mutations into the center of a PCR fragment (Higuchi et al., 1988; Ho et al., 1989; Mullis et al., 1986), making site‐directed mutagenesis more flexible. Horton et al. (1989) modified SOE by PCR to allow DNA segments from two different genes to be spliced together by overlap extension. SOE has been used to enhance site‐directed mutagenesis (Duan et al., 2013; Hussain & Chong, 2016; Xiao et al., 2007), generation of non‐polar, markerless deletions in bacteria (Kim et al., 2013; Merritt et al., 2007; Xu et al., 2013), multiple‐site fragment deletion (Zeng et al., 2017), and generation of hybrid proteins of immunological interest (Warrens et al., 1997).

*Pantoea ananatis* is considered an emerging pathogen based on the increasing number of reports of diseases occurring in various economically important crops worldwide. This pathogen can also infect humans and numerous insects (Coutinho & Venter, 2009; Dutta et al., 2016; Weller‐Stuart et al., 2017) and cause bacteremia infection (De Baere et al., 2004). *P. ananatis* PA13 causes plant diseases such as rice grain rot, sheath rot, and onion center rot disease in Korea (Choi, Kim, et al., 2012; Choi et al., 2012; Kim & Choi, 2012). This pathogen is a potential threat to stable rice production, in particular during the growing season, when the weather is hot and humid. The pathogenicity of this bacterium is controlled by bacterial quorum sensing (QS), which is bacterial cell‐to‐cell communication with extracellular signaling molecules called autoinducers that are present in the environment in proportion to cell density (Lee, 2015; Morohoshi et al., 2007; Platt & Fuqua, 2010). The QS system facilitates community coordination of gene expression and benefits group behaviors. QS of *P. ananatis*, which uses EanRI homologous to *P. stewartii* subsp. *stewartii* EsaRI, has revealed that EanR negatively regulates self‐expression and EPS production, but not *eanI* expression (Beck von Bodman & Farrand, 1995; Lee, 2015; Minogue et al., 2005; Morohoshi et al., 2007). In *P. ananatis*, 3‐oxo‐hexanoyl homoserine lactone (3‐oxo‐C6AHL) and hexanoyl homoserine lactone (C6AHL) signals are generated by EanI and secreted extracellularly. AHL signals bind EanR, an AHL receptor; this interaction de‐represses the EanR negative regulator (Morohoshi et al., 2007). We revealed that EPS production, the hypersensitive response in tobacco, and virulence in rice are regulated by AHL‐mediated QS in *P. ananatis* PA13 (Lee, 2015). In a previous study by Morohoshi et al. (2007), the QS system of onion pathogenic *P. ananatis* regulated EPS biosynthesis, biofilm formation, and infection of onion leaves.

It is established that Hfq and sRNAs are important regulators of virulence in the phytopathogen *P. ananatis* (Kang, 2017; Shin et al., 2019). The RNA chaperone Hfq and sRNAs are important regulators of virulence in *P. ananatis* (Kang, 2017; Shin et al., 2019). Hfq, a ring‐shaped hexameric RNA binding protein, has many important physiological roles that are mediated by interaction with Hfq‐dependent small RNAs (sRNAs) in bacteria (Brennan & Link, 2007). Hfq was first reported in *Escherichia coli* as a host factor important in the replication of bacteriophage Qβ (Muffler et al., 1996). Hfq regulates the stress response protein RpoS, which controls many stress response genes (Brown & Elliott, 1996; Hwang et al., 2011; Mandin & Gottesman, 2010); it also regulates virulence in several pathogenic bacteria (Chao & Vogel, 2010; Sittka et al., 2007; Zeng et al., 2013). Also, it modulates a wide range of physiological responses in bacteria. The *hfq* deletion mutant exhibits several different phenotypes (Figueroa‐Bossi et al., 2006). The Hfq protein interacts with A/U‐rich regions of untranslated sRNAs of 50–250 nucleotides with tree stem–loop sequence motifs (Lorenz et al., 2010) and assists with sRNA base pairing with target mRNA (Beisel & Storz, 2010) and the regulation of gene expression (Bardill & Hammer, 2012; Fröhlich & Vogel, 2009; Vogel & Wagner, 2007). Hfq is required for the functioning of several regulatory sRNAs, including OxyS and RyhB (Aiba, 2007; Gaida et al., 2013; Majdalani et al., 2005; Storz et al., 2004). sRNAs act as activators or repressors of protein translation through complementary base pairing with mRNA in response to changes in environmental conditions (Beisel & Storz, 2010; Gottesman et al., 2006; Waters & Storz, 2009). Several sRNAs regulate RpoS, including ArcZ. ArcZ (also called RyhA and SraH) binds Hfq and positively regulates regulatory RNA, which controls the translation of RpoS (Repoila et al., 2003). ArcZ also regulates virulence, exopolysaccharide (EPS) production, and motility in bacterial plant pathogens (Bak et al., 2014; Papenfort et al., 2009; Schachterle & Sundin, 2019; Soper et al., 2010; Zeng & Sundin, 2014).

It is established that the Hfq^ArcZ^ →RpoS Ͱ ClpXP regulatory networks are based on *E. coli*. RpoS is positively regulated by Hfq and its cognate sRNA ArcZ. RpoS levels are kept low by constitutive degradation of the ClpXP protease until the stationary phase (Raju et al., 2012). RpoS‐dependent carotenoid production in *P. agglomerans* (formerly *Erwinia herbicola*) has been previously reported (Becker‐Hapaka et al., 1997). In this study, we therefore investigated how QS, Hfq, and RpoS are involved in and regulate the carotenoid production in *P. ananatis*. Here, we also found that carotenoids were responsible for toxoflavin tolerance in *P. ananatis*.

## 2 EXPERIMENTAL PROCEDURES

### 2.1 Bacterial strains and plasmids

Bacterial strains and plasmids used in this study are listed in Table A1. *E. coli* strains were cultured on the lysogeny broth (LB) medium at 37°C. The *P. ananatis* PA13 was cultivated at 28°C on LB medium. Antibiotics were used at the following concentrations: ampicillin, 100 µg/ml; kanamycin, 50 µg/ml; rifampicin, 50 µg/ml; tetracycline, 10 µg/ml; and gentamycin, 25 µg/ml. 5‐Bromo‐4‐chloro‐3‐indoyl‐b‐D‐galactopyranoside (X‐gal) was used at 40 µg/ml when necessary.

### 2.2 DNA manipulation and data analyses

Manipulation of genomic DNA and plasmids and DNA cloning were performed as previously described (Sambrook & Russell, 2001). Restriction enzymes (TaKaRa) were used for DNA digestion and modification. DNA sequencing was performed by Macrogen (Seoul). DNA sequences were analyzed using the BLAST program at the National Center for Biotechnology Information (Gish & States, 1993), MEGALIGN (DNASTAR, Madison, WI, USA), and GENETYX‐WIN software (Genetyx).

### 2.3 Carotenoid genes

**FIGURE 1:**
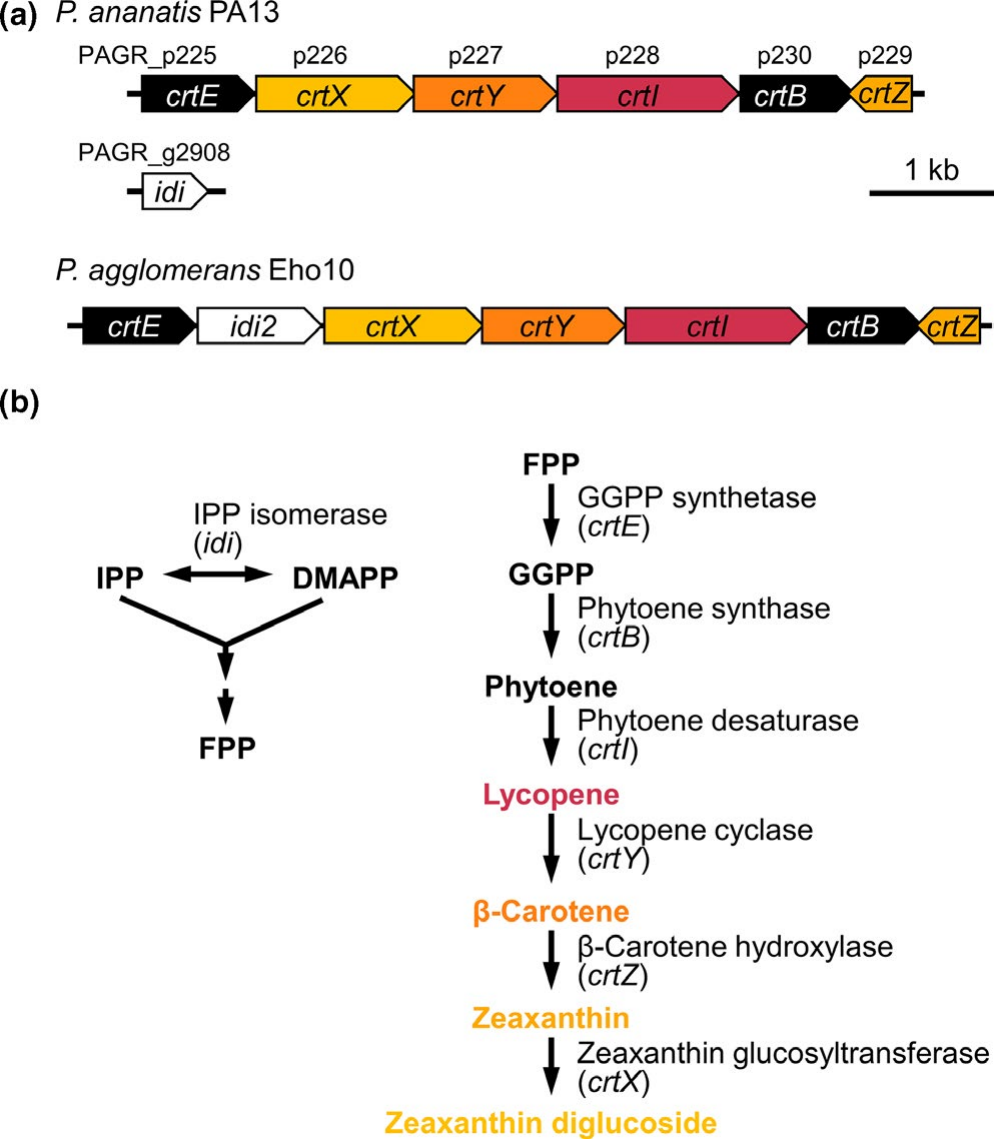
Genetic map and putative pathway responsible for carotenoid biosynthesis by *P. ananatis* PA13 (CP003086) and *P. agglomerans* Eho10 (M87280). (a) The carotenoid gene cluster of *P. ananatis* consisted of *crtE*–*X*–*Y*–*I*–*B* and *Z* and that for *P. agglomerans* of *crtE*–*idi*–*crtX*–*Y*–*I*–*B* and *Z*. Gene numbers were shown on the carotenoid gene map. (b) The putative carotenoid biosynthetic pathway of *P. ananatis* inferred according to the pathway of *Pantoea* species (Misawa et al., 1995) and plants (Guerinot, 2000)

Genomic DNA of *P. ananatis* PA13, a bacterial pathogen of rice, was used as the template to amplify carotenoid biosynthetic genes. The carotenoid genes are located on a plasmid (PAGR_p; CP003086). Figure 1 shows the carotenoid gene clusters of *P. ananatis* PA13 and *P. agglomerans* Eho10 (M87280; Hundle et al., 1994).

### 2.4 Strategy for carotenoid gene reassembly

**FIGURE A1:**
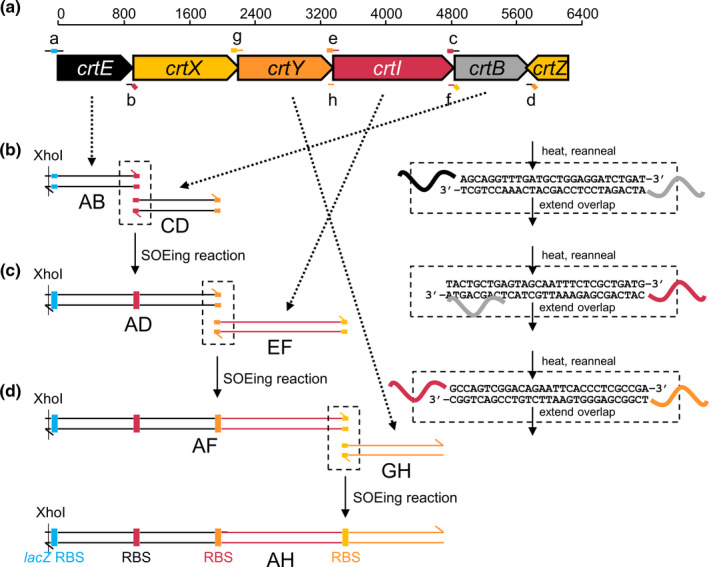
Strategy for generating recombinant carotenoid genes responsible for synthesizing phytoene, lycopene, and β‐carotene. PCR products with their overlapping regions aligned and the final rearrangement products are shown. The SOE by PCR products AD, AF, and AH are shown. In each case, the overlapping region between the primers, and the priming region in which each primer recognizes its template, was designed to have the ribosome binding sequence (RBS) of each gene. The XhoI recognition sequence and *lacZ* RBS were introduced at the beginning of SOE‐AB products. Dotted arrows indicate the rearrangements of carotenoid genes

The reassembly of the carotenoid genes responsible for synthesizing phytoene, lycopene, and β‐carotene was performed as described previously (Horton et al., 1995). The sequences of the eight primers used for reassembly are listed in Table A2. Primers “a” and “d,” “a” and “f,” and “a” and “h” are the flanking primers for PCR amplification of the final reassembled products. Primers “b” and “c,” “d” and “e,” and “f” and “g” are the SOE primers. Bases have been added to the 5′ ends of the primers in each pair to render them complementary. All of the complementary sequences have been added between primers b‒c, d‒e, and f‒g. During SOE, the upper strands of AB and the lower strands of CD overlap to act as primers (Figure A1). Fragment AB was PCR amplified from *crtE*, and fragment CD from *crtB*. Fragment EF was PCR amplified from *crtI*, and fragment GH from *crtY*. The SOE primers “b” and “c” were used to modify the PCR products of the two sequences to have an identical sequence (Table A2). Figure A1b shows the reassembly of *crtE*–*B* genes for phytoene biosynthesis. The upper strands of AB and the lower strands of CD overlap to act as primers when the PCR products are mixed, denatured, and reannealed during PCR. Fragments of AD are formed when this overlap is extended by a polymerase. Figure A1c shows the *crtE*–*B*–*I* gene reassembly for lycopene biosynthesis in which the upper strands of AD and the lower strands of EF overlap to act as primers when the PCR products are mixed, denatured, and reannealed during PCR. Fragments of AF are formed when this overlap is extended by the polymerase. Similarly, the upper strands of AF and the lower strands of GH overlap to act as primers when the PCR products are mixed, denatured, and reannealed during PCR. Fragments of AH are formed when this overlap is extended by the polymerase (Figure A1d). The XhoI recognition sequence and *lacZ* RBS were introduced at the beginning of the SOE‐AB products.

### 2.5 SOE by PCR

SOE by PCR was carried out using a T100 Thermal Cycler (Bio‐Rad, Hercules, CA, USA) for 20 cycles, each 1 min at 98°C, 1 min at 55°C, and 2 min at 70°C. The reaction was carried out in a 50 µl volume containing 2.5 U Phusion High‐Fidelity DNA polymerase (Pfu; Thermo Fisher Scientific), 200 µM dNTPs, 1 µl of primer mix (1.5 pmol per primer), and 5 µl of 10× Pfu buffer.

### 2.6 Purification and cloning of SOE fragments

The SOE products for use as templates were purified by electrophoresis in agarose (0.8% agarose, Promega) in the TAE buffer (40 mM Tris‐acetate, 1 mM ethylenediaminetetraacetic acid) with 0.5 µg/ml ethidium bromide. DNA was recovered from the gel fragment using a DNA Purification Kit (GeneAll, Seoul, South Korea). The final recombinant products were gel‐purified before cloning.

The SOE products were TA‐cloned into pGEM‐T Easy (Promega) and sequenced by Macrogen Services (Daejeon, South Korea). Error‐free clones were digested with Xhol and Sacl and ligated into the corresponding positions in pBBR1MCS5 (Kovach et al., 1995).

### 2.7 RT‐PCR analysis of wild‐type *P. ananatis* carotenoid cluster genes

Wild‐type *P. ananatis* PA13 was grown in LB medium to exponential growth phase (12 h after inoculation); total RNA was isolated using an RNeasy Mini Kit according to the supplier's instructions (Qiagen); the RNA samples were treated with RQ1 DNase (Promega) to remove any contaminating DNA. RT‐PCR was performed according to a previous report (Kim et al., 2004) as follows. Total RNA from *P. ananatis* PA13 was reverse transcribed into cDNA using M‐MLV reverse transcriptase as described by the manufacturer (Promega) at 50°C for 1 h, followed by 5 min at 75°C. Next, PCR was performed using a T100 Thermal Cycler (Bio‐Rad) under the following conditions: 96°C for 2 min, followed by 40 cycles of 96°C for 1 min, 50°C for 1 min, and 72°C for 1 min. The following primers were used for RT reactions, RT1 (*crtB*), and RT2 (*crtZ*). The following PCR primers were used: PCR1f and PCR1r; PCR2f and PCR2r; PCR3f and PCR3r; PCR4f and PCR4r; and PCR5f and PCR5r (Table A3). Southern hybridization and DNA sequencing were carried out to confirm the RT‐PCR products. As a positive control, pCOK218 DNA was used. As a negative control, PCR reactions with the same primer sets were performed using RNA samples that had not been reverse transcribed.

### 2.8 RT‐PCR analysis of reassembled carotenoid cluster genes

**FIGURE A2:**
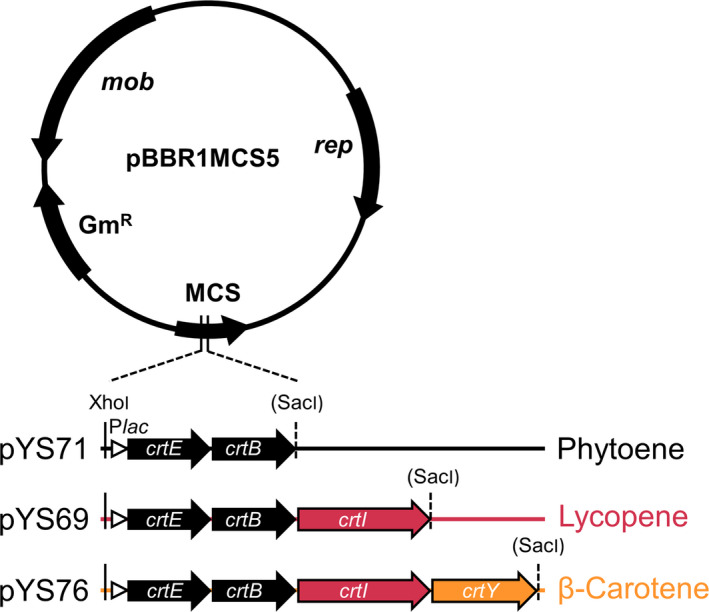
Recombinant plasmids for rearrangement of the carotenoid genes responsible for synthesizing phytoene, lycopene, and β‐carotene. The SOE by PCR products were first cloned into pGEM‐T Easy, digested with Xhol and Sacl, and ligated into the corresponding position of pBBR1MCS5. Open triangles indicate the *lacZ* RBS. The SacI site, which is dotted and parenthesized, was from the pGEM‐T Easy vector

Each SOE product was TA‐cloned into pGEM‐T Easy vector (Promega, Madison, WI, USA) and sequenced to confirm the presence of the DNA sequences (Macorgen Inc., Daejeon, South Korea). Next, clones containing *crtEB*, *crtEBI*, or *crtEBIY* were digested with Xhol and Sacl and ligated into the corresponding positions of pBBR1MCS5 (Kovach et al., 1995), generating pYS71, pYS69, or pYS76, respectively (Figure A2).

*Escherichia coli* harboring recombinant pYS71, pYS69, or pYS76 was grown in LB medium to exponential growth phase (12 h after inoculation). The following primers were used for RT reactions: RT3 (*crtZ*); RT4 (*crtI*); and RT5 (*crtY*). The following PCR primers were used: PCR6f and PCR6r; PCR7f and PCR7r; and PCR8f and PCR8r (Table A3). Southern hybridization and DNA sequencing were carried out to confirm the RT‐PCR products. As positive controls, pYS71, pYS69, and pYS76 were used. As a negative control, PCR reactions with the same primer sets were performed using RNA samples that had not been reverse transcribed.

### 2.9 HPLC

For carotenoid extraction and HPLC analysis, transformed *E. coli* DH5α harboring pYS71, pYS69, or pYS76 was cultured in 250 ml flasks containing 50 ml of LB broth with 25 μg/ml gentamicin at 37°C for 24 h. After centrifugation at 10,000 *g* for 10 min, the cultured cells were repeatedly extracted with 3 ml of acetone for lycopene and β‐carotene or ethanol for phytoene until the color was completely lost. The extracted solution was centrifuged and filtered through a GHP membrane (0.45 μm pore size). HPLC was performed using 20 μl of a prepared sample, with solvent A (60% acetonitrile, 38% ethyl acetate, 2% acetic acid) and solvent B (100% methanol) as the mobile phase, on a C_18_ Shim‐pack GIS‐DOS column (4.6 × 250 mm, 5 μm; Shimadzu) as a fixed phase at a flow rate of 1.5 ml/min. β‐Carotene was measured at 450 nm using a photodiode array detector. Lycopene was measured at 470 nm and phytoene at 280 nm. Phytoene, lycopene, and β‐carotene standards were purchased from Sigma‐Aldrich, Inc.

### 2.10 Generation of *lacZY*‐integrations and non‐polar deletion mutants

*lacZY* transcriptional integration mutagenesis (Campbell insertion) was performed as previously reported (Xu et al., 2013). An internal DNA fragment of *eanI* was amplified with EanI‐1E‐1 and EanI‐2 K (Table A3). The partial *eanI* fragment was purified, cloned into pGEM‐T Easy (Promega), and confirmed by sequencing. For recombinational mutagenesis, the EcoRI/KpnI‐digested *eanI* fragment was cloned into the pVIK112 suicide vector (Kalogeraki & Winans, 1997), creating pCOK153. The parent strain PA13 was conjugated with pCOK153, and kanamycin‐resistant colonies were selected. The mutants were confirmed by PCR using a primer that anneals upstream of the truncated fragment and the primer LacFuse followed by sequencing. We constructed *rpoS* and *crtE* null mutants using the same method as described previously.

Non‐polar deletion mutagenesis was performed as previously reported (Xu et al., 2013). We amplified upstream and downstream fragments (approximately 450 bp) of the targeted gene region by PCR using the corresponding primer pairs (Table A3). After purification, the fragments were fused by overlap PCR. The final PCR products were cloned into pGEM‐T Easy and confirmed by DNA sequencing. The fragments were excised using appropriate restriction enzymes and ligated into the suicide vector pNPTS138‐R6 KT (Lassak et al., 2010). The resulting plasmids were introduced into PA13 by conjugative mating, and mating cells were spread on an LB medium containing kanamycin and rifampicin. Single‐crossover integrates were selected on LB plates containing kanamycin and rifampicin. Single colonies were grown overnight in LB with rifampicin (25 μg/ml) and plated on LB containing 5% (w/v) sucrose to select for plasmid excision. We checked kanamycin‐sensitive colonies for targeted deletion with colony PCR using primers bracketing the location of the deletion.

### 2.11 Gene complementation

To generate target gene complementary strains, we cloned each intact target gene into the broad host range plasmid vectors pBBR1MCS5 (Kovach et al., 1995), pSRKGm (Khan et al., 2008), or pLAFR3 (Keen et al., 1988), generating pCOK218 (pBBR1MCS5::*crtEXYIBZ*), pCOK197 (pBBR1MCS5::P*_lac_*‒*eanI*), pCOK199 (pBBR1MCS5::P*_lac_*‒*eanR*), pCOK312 (pSRKGm::P*_lac_*‒*rpoS*), pBS28 (pLAFR3::*hfq*, pLAFR3::*arcZ*), or pOR78 (pBBR1MCS5:: P*_lac_*‒*clpP*) which were transferred to the corresponding mutant strains by conjugation (Table A1).

### 2.12 Toxoflavin and UV radiation tolerance

Overnight cultures of the PA13 derivatives were sub‐cultured and grown for an additional 12 h. A 100‐μl aliquot was removed, and serially diluted 10‐fold and 10‐μl of each culture was spotted on LB agar plates supplemented with 20 μg/ml toxoflavin. The spotted plates were incubated at 28°C for 36 h.

For the UV radiation tolerance assays, PA13 derivatives were spotted on LB plates using the above procedure and treated as previously described (Mohammadi et al., 2012).

### 2.13 Carotenoid production

To determine the carotenoid content of cells, *P. ananatis* strains were grown in 5 ml of LB medium at 28°C. Cells were harvested by centrifugation at 10,000 *g* for 1 min and suspended in 1 ml of methanol. The samples were vortexed for 10 min and centrifuged at 10,000 *g* for 10 min, and the methanol supernatant containing carotenoids was transferred to a new tube. We quantified the carotenoid content of the extracts by measuring the absorbance at 450 nm using a Genesys 10S UV‐VIS spectrophotometer (Thermo Fisher Scientific).

### 2.14 AHL signal assay

The isolation and purification of AHLs were performed as described by Kim et al. (2004). The culture supernatants from time course cultures of *P. ananatis* PA13 and mutants were extracted with ethyl acetate (1:1). The ethyl acetate layer was dried, and the residue was dissolved in methanol. The ethyl acetate extracts were applied to C_18_ reversed‐phase TLC plates (Merck) and developed with 60% methanol. The TLC plates were dried in a fume hood and overlaid with soft agar containing *Chromobacterium violaceum* CV026 cells cultured overnight. The plates were incubated at 28°C overnight.

### 2.15 β‐Galactosidase assay

We generated non‐polar deletions of *lacZY* genes from wild‐type PA13 named PA13L and confirmed that all traits were identical in the two strains. Wild‐type and mutant backgrounds used in the β‐galactosidase assays were PA13L. All of the test strains were grown for 20 h and sub‐cultured in LB broth at 28°C. The assays were performed using exponential‐phase cultures at an OD_600_ of ~0.4 as described previously (Choi et al., 2019).

## 3 RESULTS

### 3.1 Identification of the carotenoid biosynthetic gene cluster in *P. ananatis* PA13

We previously reported the whole genome sequence of *P. ananatis* PA13 (Choi, Lim, et al., 2012), which revealed a carotenoid gene cluster on a plasmid (PAGR_p; CP003086). Figure 1 shows the genetic map and putative pathway responsible for carotenoid biosynthesis in *P. ananatis* PA13. The open reading frames (*orfs*) in the carotenoid biosynthetic gene cluster were analyzed and annotated as *crtE*, *crtX*, *crtY*, *crtI*, *crtB*, and *crtZ* in sequence. When comparing the *crt* gene clusters between *P. ananatis* and the genetically close species *P. agglomerans*, there is a significant difference in the position of *idi*, which is located in the chromosome in the former strain (PAGR_g2908) and between *crtE* and *crtX* in the latter. The structure of the other genes is identical in the two strains (Figure 1a).

The putative carotenoid biosynthetic pathway of *P. ananatis* was inferred from the pathways of *Pantoea* species (Hundle et al., 1994; Misawa et al., 1995) and plants (Guerinot, 2000). Carotenoid biosynthesis begins with isomerization of isopenthyl diphosphate (IPP) from the mevalonate pathway to produce dimethylallyl diphosphate (DMAPP) in a reaction catalyzed by IPP isomerase encoded by *idi*. Carotenoids are produced from the common precursor farnesyl diphosphate (FPP). The addition of a further IPP molecule yields geranylgeranyl diphosphate (GGPP) in a reaction catalyzed by GGPP synthetase (encoded by *crtE*). The next step in the carotenoid pathway is the head‐to‐head condensation of two molecules of GGPP to produce phytoene in a reaction catalyzed by phytoene synthase (encoded by *crtB*). Sequentially, the involved enzymes include phytoene desaturase (encoded by *crtI)*, lycopene β‐cyclase (*crtY*), β‐carotene hydroxylase (*crtZ*), and zeaxanthin glucosyltransferase (*crtX*) (Figure 1b).

### 3.2 Carotenoid biosynthetic cluster genes *crtEXYIB* of *P. ananatis* are polycistronic

**FIGURE 2:**
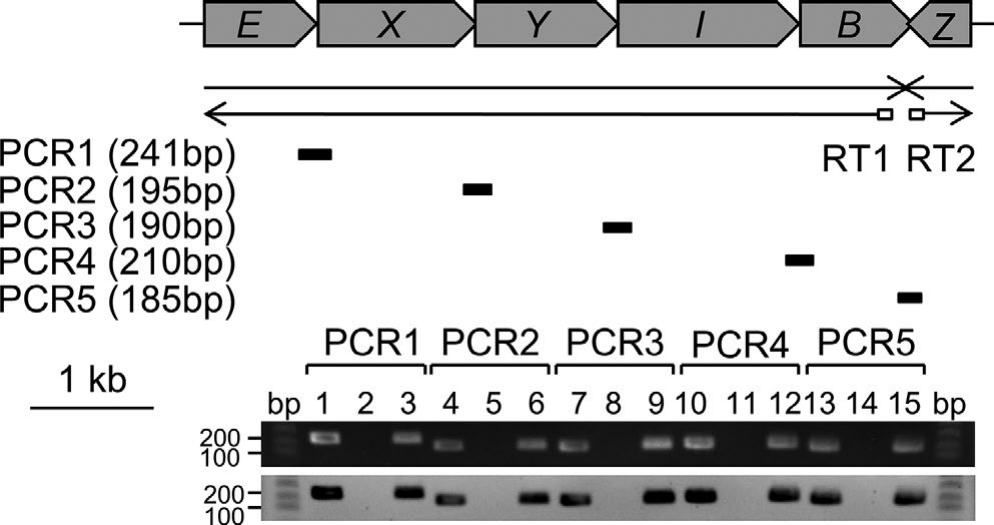
Confirmation of transcriptional units in the carotenoid gene cluster of *P. ananatis* by RT‐PCR. Black arrows indicate the extension and transcription directions of the *crtEXYIB* operon and *crtZ* gene. An arrow below the open arrows represents the product of RT reactions. The short thick bars below the RT arrow indicate the PCR products from the corresponding RT reactions. The expected sizes of the PCR products are indicated below the labels. Agarose gel analysis (upper panel) and Southern analysis (lower panel) of the RT‐PCR products of the *crtEXYIB* operon and *crtZ* gene. Lanes 1–3, 4–6, 7–9, 10–12, and 13–15 correspond to the products of PCR1, PCR2, PCR3, PCR4, and PCR5, respectively. Lanes 1, 4, 7, 10, and 13: PCR products from the DNA template as positive controls; lanes 2, 5, 8, 11, and 14: PCR products from the RNA template as negative controls; and lanes 3, 6, 9, 12, and 15: RT‐PCR products

We performed reverse transcription‐polymerase chain reaction (RT‐PCR) to determine whether the wild‐type *P. ananatis* carotenoid biosynthetic cluster genes are polycistronic. We used five sets of primers to amplify *crtE*–*X*, *X*–*Y*, *Y*–*I*, *I*–*B*, and *B*–*Z*. RT‐PCR followed by Southern hybridization indicated that the *P. ananatis* carotenoid biosynthetic cluster genes *crtEXYIB* are transcribed as a single transcript, and *crtZ* is transcribed as an independent single transcript in the opposite direction (Figure 2).

### 3.3 Cloning of SOE fragments and RT‐PCR analysis

**FIGURE A3:**
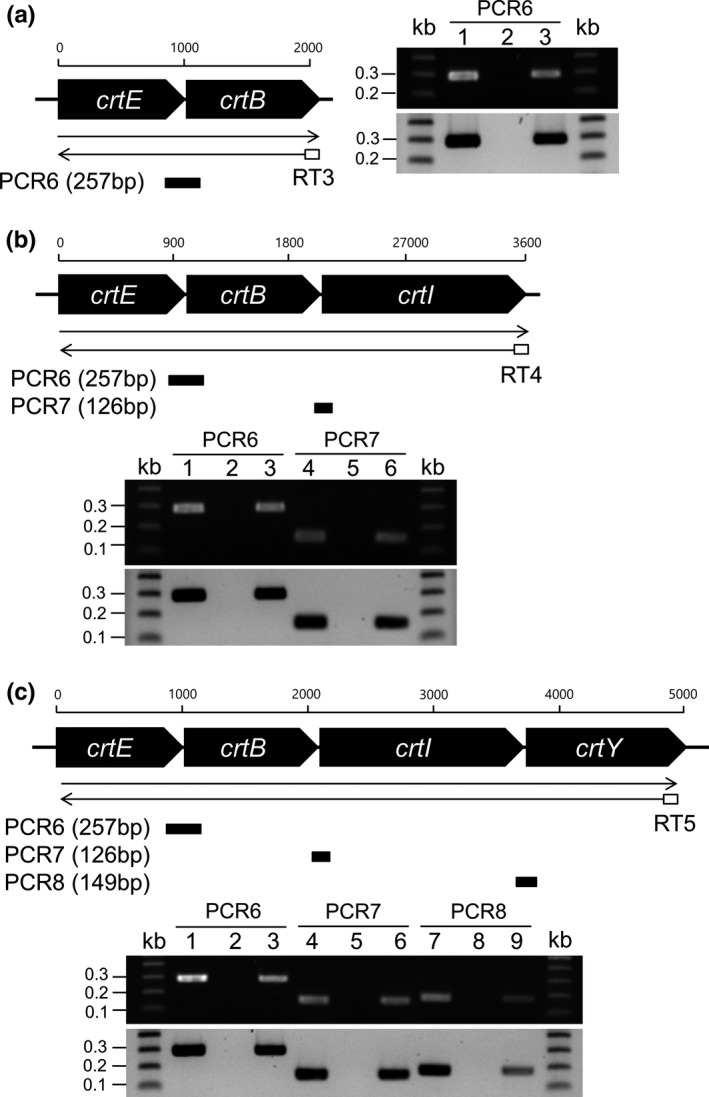
Confirmation of transcriptional units in the reassembled *crtE*–*B*, *crtE*–*B*–*I*, and *crtE*–*B*–*I*–*Y* operons by RT‐PCR. RT‐PCR products were confirmed by Southern hybridization. Black arrows indicate the extension and transcription directions of the *crtE*–*B*, *crtE*–*B*–*I*, and *crtE*–*B*–*I*–*Y* operons on plasmids pYS71 (a), pYS69 (b), and pYS76 (c), respectively. Arrows below the open arrows represent the products of RT reactions. The short thick bars below the RT arrow indicate the PCR products from the corresponding RT reactions. The expected sizes of the PCR products are indicated below the labels. Agarose gel analysis (upper panel) and Southern analysis (lower panel) of the RT‐PCR products of the *crtE*–*B*, *crtE*–*B*–*I*, and *crtE*–*B*–*I*–*Y* operons. Southern hybridization was performed using the *crtE*–*B*–*I*–*Y* operon region (2.2, 3, and 5 kb XhoI–SacI fragments of pYS71, pYS69, and pYS76, respectively) as probes. Lanes 1–3, 4–6, and 7–9 correspond to the products of PCR1, PCR2, and PCR3, respectively. Lanes 1, 4, and 7: PCR products from the DNA template as positive controls; lanes 2, 5, and 8: PCR products from the RNA template as negative controls; and lanes 3, 6, and 9: RT‐PCR products

We used RT‐PCR to determine whether the reassembled *crtEB*, *crtEBI*, and *crtEBIY* clones on the plasmids pYS71, pYS69, and pYS76 (Figure A2) are transcribed as a single transcript. We used three sets of primers to amplify *crtE*–*B*, *B*–*I*, and *I*–*Y*. RT‐PCR followed by Southern hybridization indicated that the reassembled *crtE*–*B*, *crtE*–*B*–*I*, and *crtE*–*B*–*I*–*Y* clones on the plasmids pYS71, pYS69, and pYS76 are transcribed as single transcripts (Figure A3).

### 3.4 Carotenoid production in *E. coli*

**FIGURE 3:**
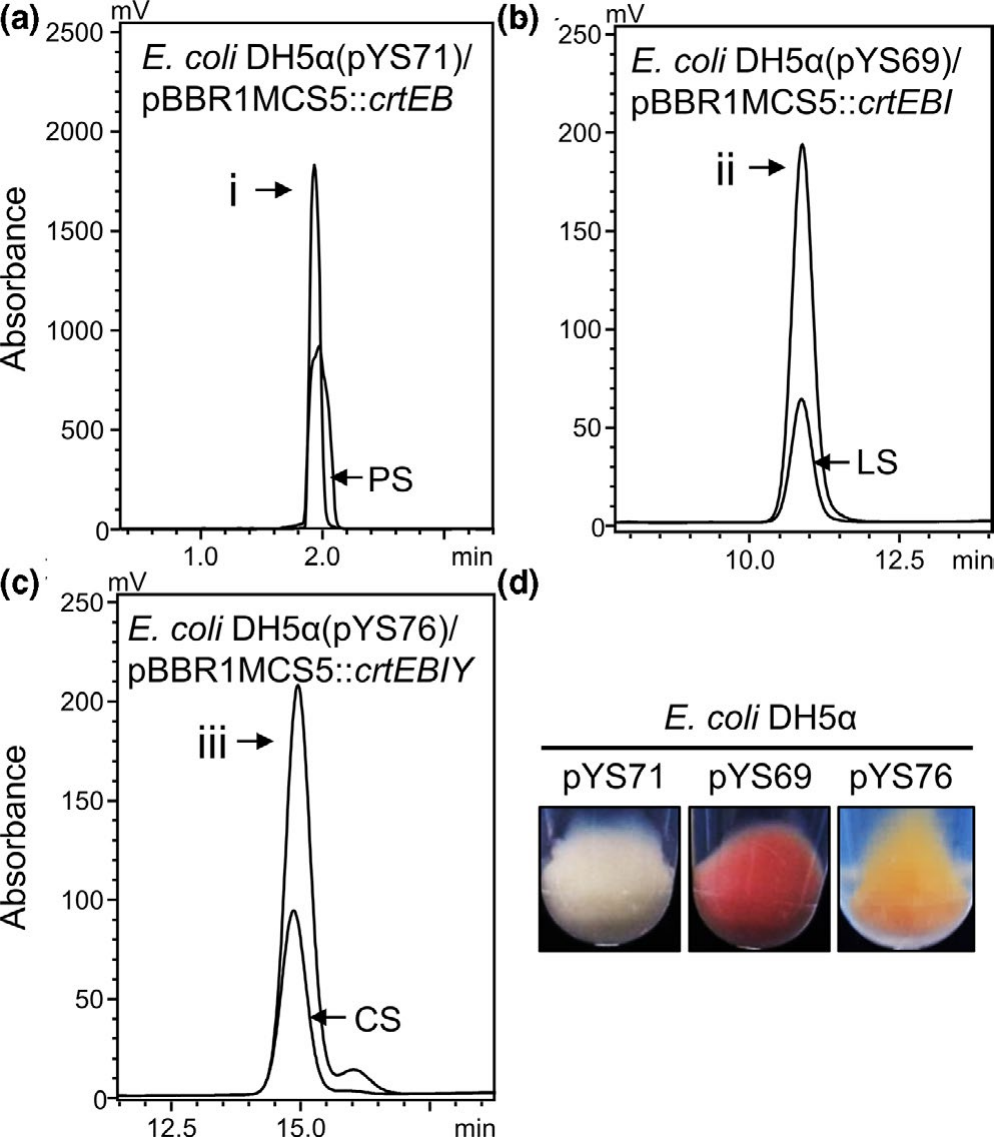
Production of phytoene, lycopene, and β‐carotene in *E. coli*. HPLC analysis confirmed that the *E. coli* strains harboring pYS71, pYS69, and pYS76 produced phytoene (a), lycopene (b), and β‐carotene (c); respectively. i, *E. coli* DH5α/pYS71(pBBR1MCS5::*crtE*–*B*) producing phytoene (retention time 2 min, 280 nm); ii, *E. coli* DH5α/pYS69(pBBR1MCS5::*crtE*–*B*–*I*) producing lycopene (retention time 11 min, 470 nm); and iii, *E. coli* DH5α/pYS76(pBBR1MCS5::*crtE*–*B*–*I*–*Y*) producing β‐carotene (retention time 14.8 min, 450 nm). PS, LS, and CS indicate the phytoene, lycopene, and β‐carotene standards, respectively. (d) The color change of harvested *E. coli* cells harboring pYS71, pYS69, or pYS76. The harvested cells showed colorless phytoene, magenta lycopene, or orange β‐carotene

To determine whether *E. coli* DH5α transformed with pYS71/pSRKGm::*crtE*–*B*, pYS69/pSRKGm::*crtE*–*B*–*I*, or pYS76/pSRKGm::*crtE*–*B*–*I*–*Y* produces phytoene, lycopene, or β‐carotene, respectively, we performed high‐performance liquid chromatography (HPLC). The results revealed that *E. coli* DH5α/pYS71/pSRKGm::*crtE*–*B* produced colorless phytoene, as confirmed by the standard peak at the same retention time (Figure 3a,d); *E. coli* DH5α/pYS69/pSRKGm::*crtE*–*B*–*I* produced magenta lycopene, as confirmed by the standard peak at the same retention time (Figure 3b,d); and *E. coli* DH5α/pYS76/pSRKGm::*crtE*–*B*–*I*–*Y* produced orange β‐carotene, as confirmed by the standard peak at the same retention time (Figure 3c,d). SOE enabled the reassembly of multiple carotenoid synthetic genes and the production of carotenoids in *E. coli*.

### 3.5 Carotenoid confers *P. ananatis* with tolerance to toxoflavin and UV radiation

**FIGURE 4:**
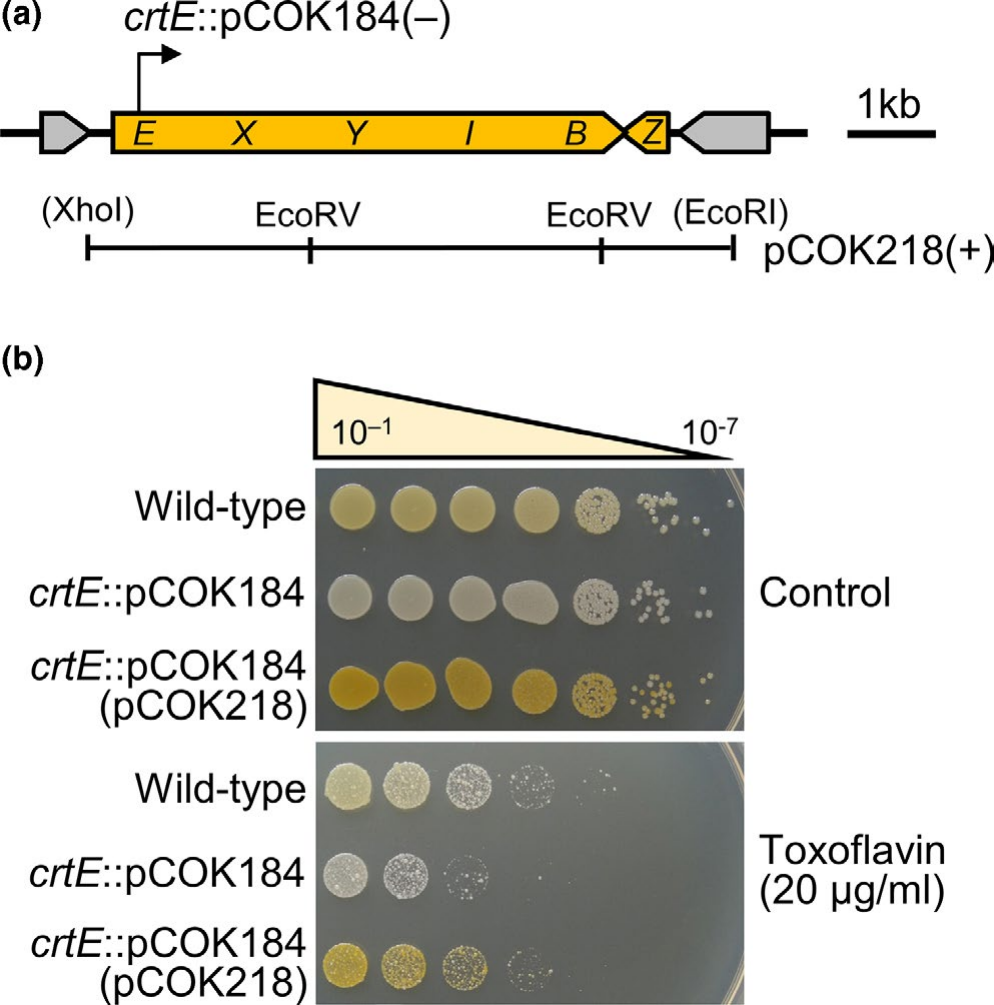
Carotenoids confer toxoflavin tolerance to *P. ananatis*. (a) Construction of the *crtE*::pCOK184 mutant and complementation plasmid pCOK218. − or + indicates negative or positive carotenoid production, respectively. (b) Toxoflavin tolerance of *P. ananatis*. The wild‐type and *crtE*::pCOK184 mutant carrying pCOK218 exhibited greater toxoflavin tolerance than the *crtE* mutant; however, the *crtE*::pCOK184 mutant was more sensitive than the wild‐type to toxoflavin at 20 µg/ml. *Pantoea ananatis* PA13 is sensitive to toxoflavin concentrations >20 µg/ml

**FIGURE A4:**
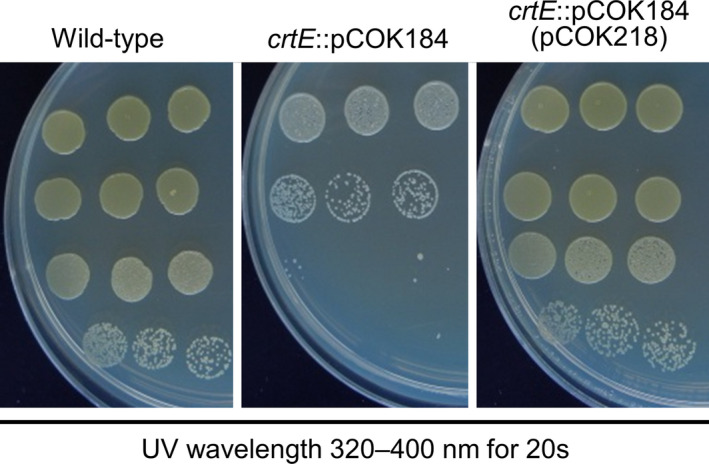
Carotenoid production confers UV radiation tolerance to *P. ananatis*. The wild‐type and *crtE*::pCOK184 mutant harboring pCOK218 exhibited greater UV radiation tolerance than the *crtE* mutant at wavelengths of 320–400 nm for 20 s; however, the *crtE*::pCOK184 mutant showed lower UV radiation tolerance than the wild‐type

Toxoflavin is a phytotoxin produced by *B*. *glumae*, a rice grain pathogen that shares rice environments with *P. ananatis* and has antibacterial properties. To determine whether the carotenoid production in *P. ananatis* is responsible for tolerance to toxoflavin and UV radiation, we generated a polarized *crtE*::pCOK184 mutant by Campbell insertion (Figure 4a). Complementation plasmid pCOK218 was also generated by cloning the carotenoid biosynthetic genes *crtE*–*Z* into pBBR1MCS5 (Figure 4a), which recovered the carotenoid deficiency in the *crtE*::pCOK184 mutant (Figure 4b). The wild‐type is sensitive to toxoflavin concentrations >20 µg/ml (Figure 4b). The *crtE*::pCOK184 mutant exhibited lower tolerance than the wild‐type to 20 µg/ml toxoflavin; however, the wild‐type and complementation strain (+) showed greater tolerance than the *crtE* mutant (Figure 4b). These results were consistent with those for UV radiation tolerance, but the survival of the *crtE*::pCOK184 mutant was approximately 100 times lower than that of the wild–type (Figure A4).

### 3.6 Carotenoid production depends on RpoS, which is positively regulated by Hfq/ArcZ and negatively by ClpXP in *P. ananatis*

**FIGURE 5:**
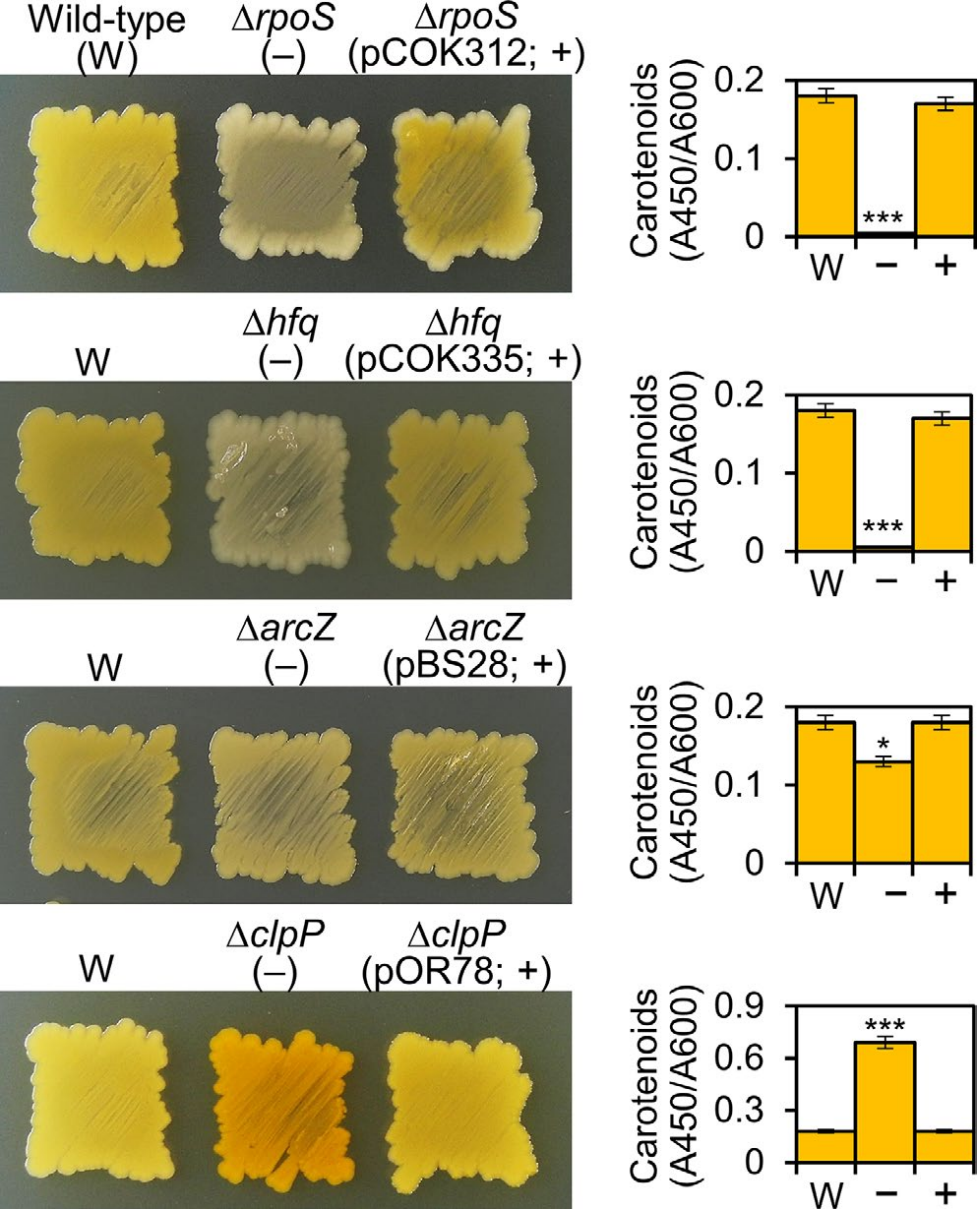
Carotenoid production in the wild‐type (W), ∆*rpoS*, ∆*hfq*, ∆*arcZ*, ∆*clpP*, and complementation (+) strains. Values are means ± standard deviation (*SD*) of three independent experiments. *p < 0.05; **p < 0.01; ***p < 0.001 vs. wild‐type

The colonies of ∆*rpoS* and ∆*hfq* mutants were white, and neither produced carotenoids (Figure 5); however, colonies of complementation strains (+) carrying pCOK312 and pCOK335, respectively, were orange and produced carotenoids. Colonies of the ∆*arcZ* mutant were faint orange and exhibited a slight reduction in carotenoid production (Figure 5), indicating involvement in carotenoid production. Colonies of the ∆*clpP* mutant were dark orange and exhibited an approximately twofold increase in carotenoid production, indicating a negative carotenoid regulation via RpoS inhibition (Figure 5). Complementation strains (+) of ∆*arcZ* and ∆*clpP* mutants carrying pBS28 and pOR78, respectively, produced amounts of carotenoids similar to that of the wild‐type. These results suggest that carotenoid production of *P. ananatis* depends on RpoS, which is positively regulated by Hfq/ArcZ and negatively by ClpP, similar to an important regulatory network of *E. coli* (Hfq^ArcZ^ →RpoS Ͱ ClpXP).

### 3.7 EanR negatively regulates carotenoid production in *P. ananatis*

**FIGURE A5:**
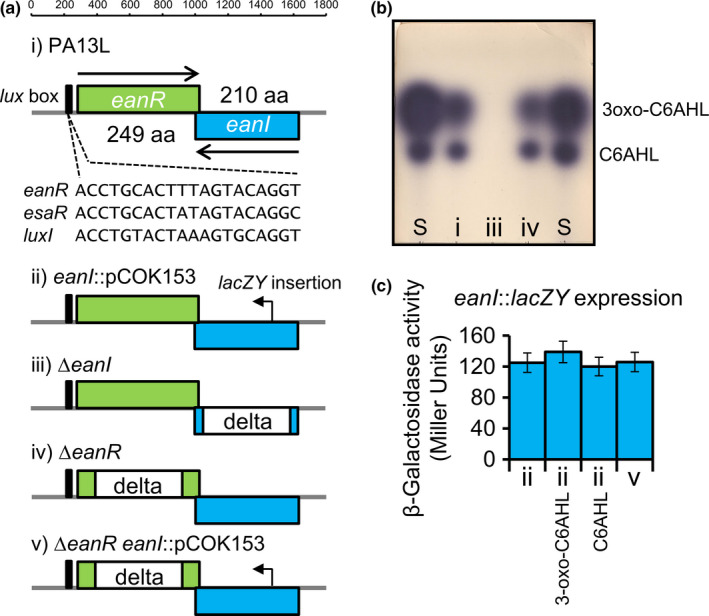
QS system of *P. ananatis* PA13. (a) Genetic map of the *eanR* and *eanI* loci of the QS system in *P. ananatis* PA13 and mutant generation. The putative *lux* box is upstream of *eanR*, for comparison, the *esaR lux* box of *P. stewartii* subsp. *stewartii* and *lux* box of *Vibrio fischeri* are presented. Campbell insertion and non‐polar deletion mutants were generated to determine if *eanR* regulates the expression of *eanI*. (b) Characterization and quantification of AHL signals of the PA13 derivatives. (c) β‐Galactosidase activity reporting *eanI* expression. (i) PA13L, non‐polar deletion of *lacZY* genes from wild‐type PA13 used in the β‐galactosidase assays as the wild‐type; (ii) *eanI*::pCOK153 (pVIK112 carrying truncated *eanI* at both ends); (iii) ∆*eanI*, non‐polar deletion of *eanI*; (iv) ∆*eanR*, non‐polar deletion of *eanR*; and (v) ∆*eanR eanI*::pCOK153. S indicates synthetic C6‐HSL and 3‐oxo‐C6‐HSL used as AHL standards

A previous report examined EanR de‐repression in the QS system of *P. ananatis*, which causes center rot disease in onion (Morohoshi et al., 2007). QS of *P. ananatis* PA13 is also similar to that of *P. stewartii* (Minogue et al., 2005). Figure A5 shows the QS system of *P. ananatis* PA13. The *eanR* and *eanI* genes are transcribed in the opposite direction, and the *lux* box is at the *eanR* gene promoter region (Figure A5a). To determine whether *eanI* expression is under the control of EanR, we constructed a *lacZY* integration of *eanI*::pCOK153 (i.e., pVIK112 carrying *eanI* truncated at both ends) in PA13L and PA13L∆*eanR* mutant backgrounds using Campbell insertion (Figure A5a). QS signal production of the mutants was confirmed using thin‐layer chromatography (TLC) and a *Chromobacterium* indicator strain. The *eanI* mutant did not produce QS signals, whereas the *eanR* mutant did (Figure A5b). The expression of *eanI* was not decreased in the ∆*eanR* mutant background or increased by the addition of 3‐oxo‐C6AHL or C6AHL (Figure A5c). These data indicate that the expression of *eanI* is not under the control of EanR.

**FIGURE 6:**
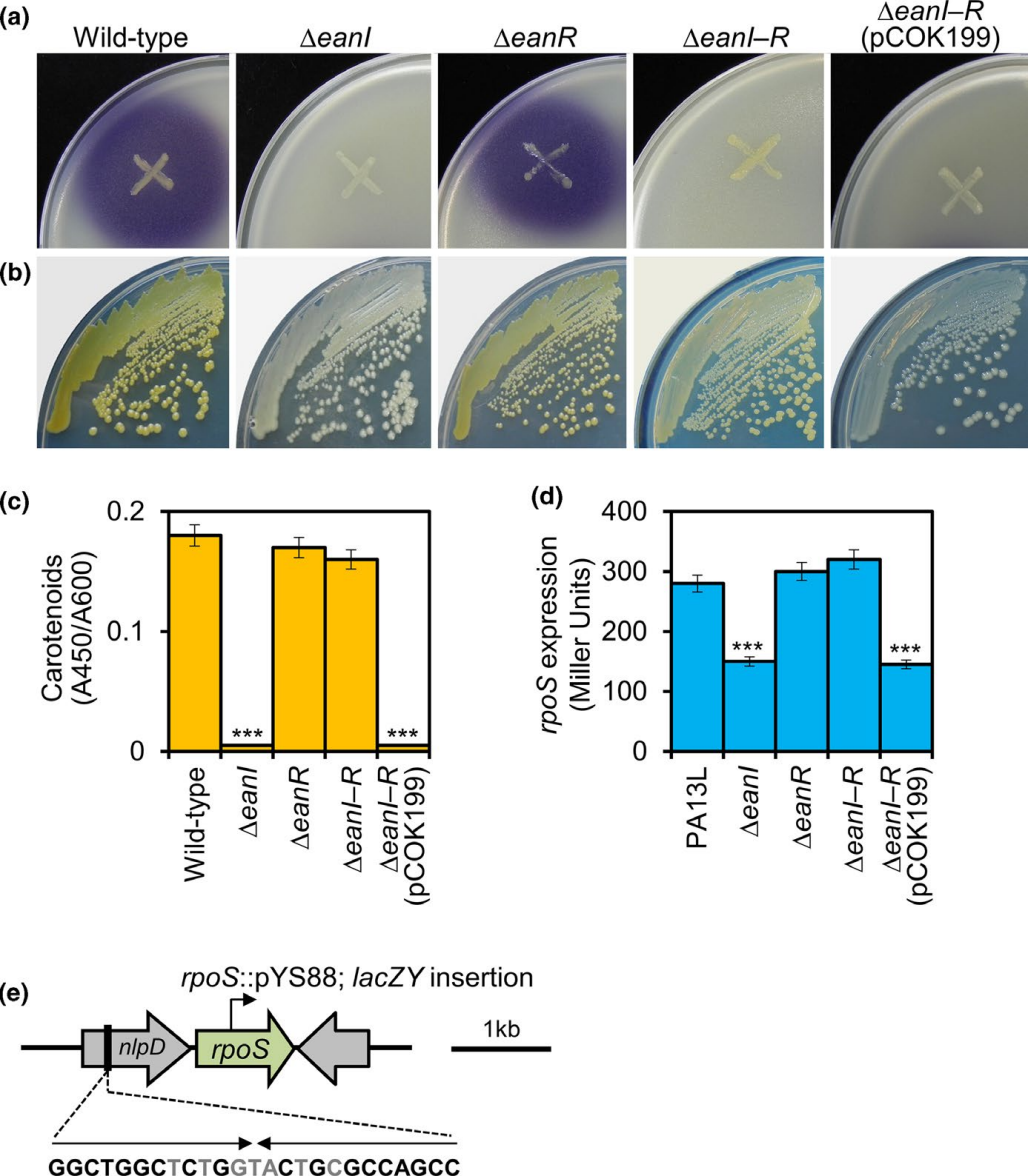
EanR negatively regulates carotenoid production *via* inhibition of *rpoS*. (a) QS signal production of the wild‐type and ∆*eanI*, ∆*eanR*, and ∆*eanI‒R* mutants as well as the ∆*eanI‒R* mutant carrying pCOK199 on *C*. *violaceum* CV026 biosensor‐embedded plates. (b) Carotenoid production of the wild‐type and ∆*eanI*, ∆*eanR*, and ∆*eanI‒R* mutants as well as the ∆*eanI‒R* mutant carrying pCOK199. (c) Quantification of carotenoid production of the PA13 derivatives. Carotenoid production was identical to that shown in (b). Values are means ± standard deviation (*SD*) of three independent experiments. ***p < 0.001 versus wild‐type. (d) β‐Galactosidase activity reporting *rpoS* expression. *rpoS* expression was induced in the absence of EanR and decreased in the absence of EanI, indicating that EanR negatively regulates *rpoS* expression and QS signals de‐repress EanR. Values are means ± standard deviation (*SD*) of three independent experiments. ***p < 0.001 versus PA13L. (e) Genetic map of *rpoS* locus and putative *lux* box. Inverted repeat sequences are shown in bold

We performed functional phenotypic de‐repression of EanR using ∆*eanI*, ∆*eanR*, and ∆*eanI‒R* mutants of *P. ananatis* PA13. The ∆*eanI* mutant exhibited no production of QS signals or carotenoids; however, ∆*eanR* and ∆*eanI‒R* mutants produced carotenoids, suggesting that EanR negatively regulates carotenoid production and that EanR‐dependent repression of carotenoid biosynthesis is alleviated by the binding of AHLs to EanR (Figure 6a−c). Carotenoid production of the ∆*eanI‒R* mutant was abolished by transformation with pCOK199 (pBBR1MCS5::P*lac*‒*eanR*), confirming that EanR negatively regulates carotenoid production (Figure 6b,c).

### 3.8 EanR negatively regulates carotenoid production via inhibition of *rpoS* in *P. ananatis*

To determine whether *rpoS* expression is regulated by EanR, we constructed a *lacZY* integration of *rpoS*::pYS88 (pVIK112 carrying *rpoS* truncated at both ends) in PA13L, PA13L∆*eanI*, PA13L∆*eanR*, and PA13L∆*eanI‒R* mutant backgrounds using Campbell insertion. The expression of *rpoS* decreased significantly in the ∆*eanI* mutant background. *rpoS* expression increased in the ∆*eanR* and ∆*eanI‒R* mutant backgrounds (Figure 6d); *rpoS* expression decreased in the presence of EanR. These results indicate that EanR negatively regulates *rpoS* expression and QS signals de‐repress EanR resulting in increased expression of *rpoS*. Although the putative *lux* box suggests that EanR binds to the promoter region of *rpoS* (Figure 6e), there is currently no direct evidence for this. We analyzed the candidate *lux* box(s) in the *crtEXYIB* gene cluster or the promoter region of the *crtZ* gene, but did not find it, but did not find it.

### 3.9 QS is delayed in the absence of Hfq

**FIGURE 7:**
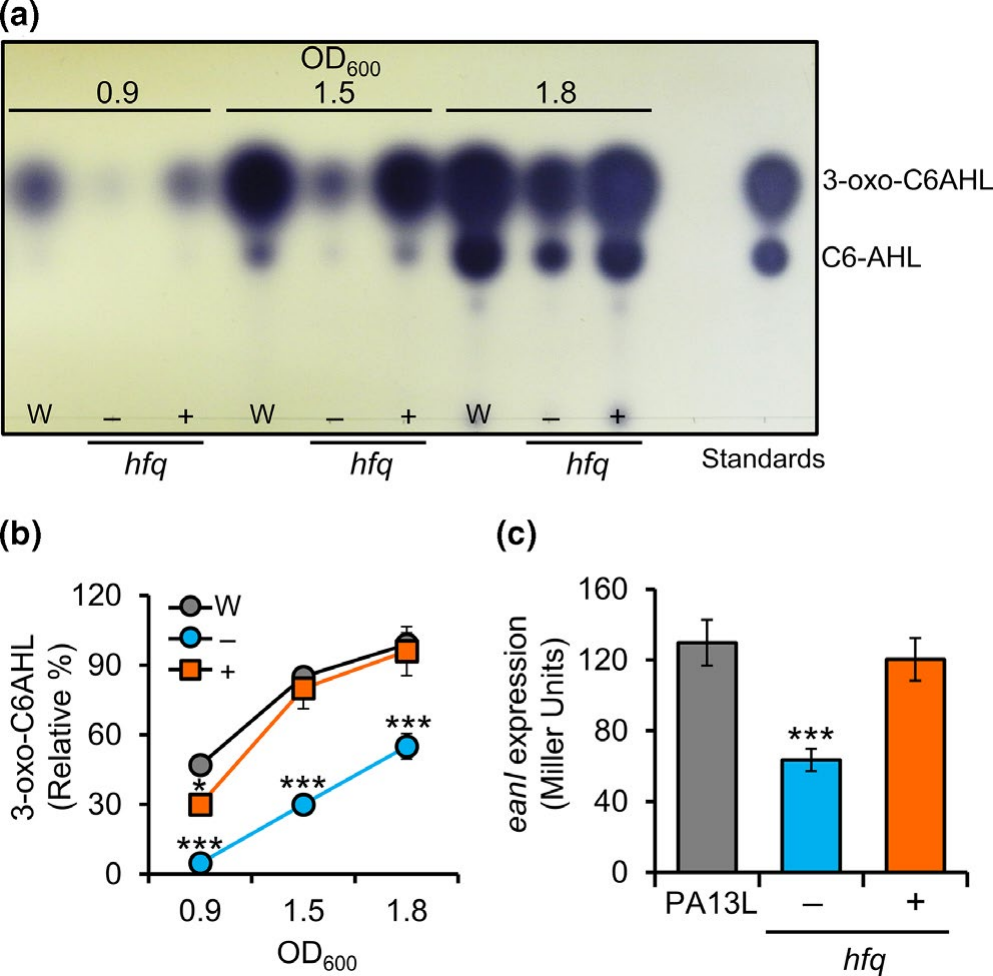
Hfq regulates the expression of *eanI* QS signal synthase. (a) Characterization and quantification of AHL signals in wild‐type (W), ∆*hfq* mutant (−), and complementation (+; pCOK335) strains of *P. ananatis* PA13. (b) 3‐oxo‐C6AHL signal production of the wild‐type (W), ∆*hfq* mutant (‒), and complementation strains carrying pCOK335 (+; pLAFR3::*hfq*). Relative percentage to the wild‐type at OD_600_ 1.8. Values are means ±standard deviation (*SD*) of three independent experiments. *p < 0.05; **p < 0.01; ***p < 0.001 versus wild‐type. (c) β‐Galactosidase activity reporting *eanI* expression in PA13L, ∆*hfq* mutant (−), and complementation strain (+; pLAFR3::*hfq*). Values are means ± standard deviation (*SD*) of three independent experiments. ***p < 0.01 versus PA13L

To elucidate the relationship between Hfq and QS, we performed QS signal‐production assays with wild‐type, ∆*hfq* mutant, and ∆*hfq* complementation strains. QS signals were extracted in the mid/late log phase (OD_600_ = 0.9, 1.5, and 1.8) and developed on C_18_ reversed‐phase TLC plates. QS signaling in the ∆*hfq* mutant (−) decreased significantly but recovered to the level of the wild‐type after transforming with pCOK335 (+; pLAFR3::*hfq*; Figure 7a,b). These results suggest that Hfq positively regulates QS signal production. We also examined whether the reduction in QS signal in the ∆*hfq* mutant was due to bacterial growth; our results showed that growth in ∆*hfq* was not retarded compared with the wild‐type strain (data not shown). Using β‐galactosidase activity assays, we found that expression of *eanI* decreased significantly in the absence of Hfq (−) but recovered by transformation with pCOK335 (+; pLAFR3::*hfq*) (Figure 7c). This is consistent with the finding that QS signal production in the ∆*hfq* mutant was significantly lower (Figure 7a,b). These results indicate that Hfq positively regulates QS, which is delayed in the absence of Hfq in *P. ananatis*.

## 4 DISCUSSION

*Pantoea ananatis* is an emerging plant pathogen that causes severe loss of many crops and trees, such as corn, onion, rice, and Eucalyptus, worldwide (Coutinho & Venter, 2009; Weller‐Stuart et al., 2017). This bacterium has also been associated with insects and humans. It is considered pathogenic because of its virulence in a wide variety of plant hosts and saprophytic ability in diverse ecological niches (Coutinho & Venter, 2009). In this study, we focused on the carotenoid biosynthesis gene cluster of *P. ananatis* and performed gene reassembly for carotenoid production. Here, we investigated the ecological and physiological functions of regulatory mechanisms of the carotenoid production of *P. ananatis*.

Much effort has been focused on the biosynthesis of carotenoids using bacterial carotenoid genes (Guerinot, 2000; Lee et al., 2003; Mijts & Schmidt‐Dannert, 2003; Misawa et al., 1995). Techniques based on recombining DNA sequences rely on restriction sites, so the primer must contain the introduced restriction site, which should not be in the center of the fragment (Higuchi et al., 1988; Ho et al., 1989; Horton et al., 1989; Mullis et al., 1986). Moreover, if multiple cloning vectors are to be used, plasmid incompatibility is also a limiting factor. Here, we applied the SOE by PCR technique to recombine DNA sequences without relying on restriction sites. In this report, we describe the reassembly of the genes encoding bacterial carotenoid biosynthetic proteins as *crtE‒B*, *crtE‒B‒I*, or *crtE‒B‒I‒Y* for the synthesis of phytoene, lycopene, or β‐carotene, respectively. *E. coli* expressing *crtE‒B*, *crtE‒B‒I*, or *crtE‒B‒I‒Y* produced phytoene, lycopene, or β‐carotene, respectively. Zeaxanthin biosynthesis was enabled by the addition of *crtZ*, but gene recombination failed despite numerous attempts. The likelihood of success decreases with an increasing number of genes to be recombined.

In practice, simply introducing *lacZ* ribosomal binding sequences (RBSs) at the beginning of the SOE‐AB product (P*lac*‒*crtE*) enables carotenoid biosynthesis. CrtE catalyzes the synthesis of GGPP, an early intermediate of carotenoid biosynthesis. We did not test whether the absolute amount of carotenoids increases as the level of GGPP increases *in vivo*.

We used a DNA template from the rice pathogenic bacterium *P. ananatis* in SOE by PCR, which is controllable and independent of restriction sequences, for the carotenoid gene reassembly. *Pantoea agglomerans*, which causes palea browning of rice, is a genetically close species to *P. ananatis* with which it shares a biological niche. The organization of the carotenoid biosynthetic gene clusters of the two strains is identical except *idi*. Interestingly, *P. agglomerans* has an *idi* gene between *crtE* and *crtX*, which distinguishes it from *P. ananatis*, suggesting that *idi* could be used to distinguish genetically similar bacteria.

SOE is a novel PCR‐mediated recombinant DNA technology that does not rely on restriction sites, so its coverage is considerably wider than standard restriction enzyme‐based methods for gene recombination. This enables finer control over recombination for genetic engineering. Besides, the sequence of the overlap region is determined by primer design, allowing simultaneous non‐polar mutagenesis, site‐directed mutagenesis, and recombination. In this study, we applied this technically simple and rapid recombinant DNA technique to the biosynthesis of three carotenoids. The technique will likely be suitable for the recombination of multiple genes.

In bacteria, carotenoids are closely related to the conditions of the surrounding environment. We found that the UV radiation tolerance of *P. ananatis* was due to the carotenoids they produce. These results are consistent with those regarding *P. stewartii* subsp. *stewartii* (Mohammadi et al., 2012). Considering the plant environment (particularly rice) in which *P. ananatis* lives, UV radiation tolerance is advantageous for survival. Interestingly, these carotenoids are unique in that they also make *P. ananatis* tolerant to toxoflavin. Toxoflavin was proposed to produce superoxide (O_2_^‒^) and H_2_O_2_ during autorecycling oxidation processes under oxygen and light (Latuasan & Berends, 1961; Nagamatsu et al., 1982). Thus, the carotenoid production in *P. ananatis* can be considered a survival strategy to reduce oxidative stress caused by toxoflavin. These results were consistent with previous studies showing that carotenoids reduce oxidation stress in bacteria (Oren, 2009; Tian & Hua, 2010). This is the first report on the production and use of carotenoids to overcome toxoflavin, resulting from *P. ananatis* and *B*. *glumae* sharing the same rice environment. Although we evaluated whether the carotenoid production of PA13 confers resistance to additional antibiotics such as ampicillin and tetracycline, the carotenoid production of PA13 did not confer tolerance to each antibiotic (data not shown).

We found that QS and Hfq are directly or indirectly involved in regulating carotenoid production in *P. ananatis* PA13. QS regulates an extensive range of functions, including bioluminescence, virulence, biofilm formation, DNA exchange, and sporulation in bacteria (Fuqua et al., 1996; Waters & Bassler, 2005). Hfq is a global RNA chaperone that interacts with sRNAs of diverse functions; it also regulates virulence and environmental stress in many plant and animal bacterial pathogens (Chao & Vogel, 2010; Ding et al., 2004; Shin et al., 2019; Zeng et al., 2013). The *hfq* mutant in *Erwinia amylovora* Ea1189 reduces virulence, amylovoran EPS production, biofilm formation, motility, and positive regulation of the type III secretion system (Zeng et al., 2013). In *Pectobacterium carotovorum*, *the hfq* mutant exhibits defects in motility, biofilm formation, sedimentation, and virulence (Wang et al., 2018). Hfq is also an important regulator of virulence, motility, and biofilm formation in *P. ananatis* LMG2665 (Shin et al., 2019). We found that Hfq regulates the expression of *eanI* encoding the QS signal synthase, which was confirmed by *eanI* expression and QS signal productivity assays. These results are consistent with the finding that Hfq regulates QS signal production directly via interactions with the AHL receptor ExpR in *Sinorhizobium meliloti* (Gao et al., 2015). QS systems integrate other global regulators, including noncoding sRNAs. This network is activated through the binding of Hfq and Hfq‐dependent sRNA and controls gene expression via post‐transcription regulation (Storz et al., 2005). There are several reports that the Hfq‐dependent sRNAs Qrr1–4 and RsmY interact with Hfq to directly and indirectly control QS targets in *Vibrio cholerae* and *Pseudomonas aeruginosa* (Kay et al., 2006; Lenz et al., 2004). Shin et al. (2019) suggested that the putative Hfq‐dependent sRNAs pPAR237 and pPAR238 are involved in regulating QS by activating EanI without genetic analyses. Further studies are needed to identify the sRNAs in *P. ananatis*. It was previously reported that EanR mediated QS regulation by de‐repression as in *P. stewartii* (Beck von Bodman & Farrand, 1995; Morohoshi et al., 2007). In *P. ananatis*, EanR represses the *ean* box (*lux* box‐like sequences) in the upstream region of *eanR* and adding AHL promoted dose‐dependent de‐repression (Morohoshi et al., 2007). This EanR‐mediated QS regulation was similar to that of the close homolog EsaR in *P. stewartii* (Minogue et al., 2005). Overall, we found that QS signal production in *P. ananatis* was delayed in the absence of Hfq since EanR negatively regulates RpoS. The expression of RpoS is entirely dependent on bacterial growth. Using EanR, *P. ananatis* must inhibit RpoS expression before reaching the stationary phase, at which point EanR is removed to initiate expression of RpoS. Hfq is responsible for determining the timing of the Hfq‐mediated increase in *eanI* expression to produce full QS signals. The resulting QS signals de‐repress EanR, followed by Hfq to express RpoS, which turns on carotenoid biosynthesis.

**FIGURE 8:**
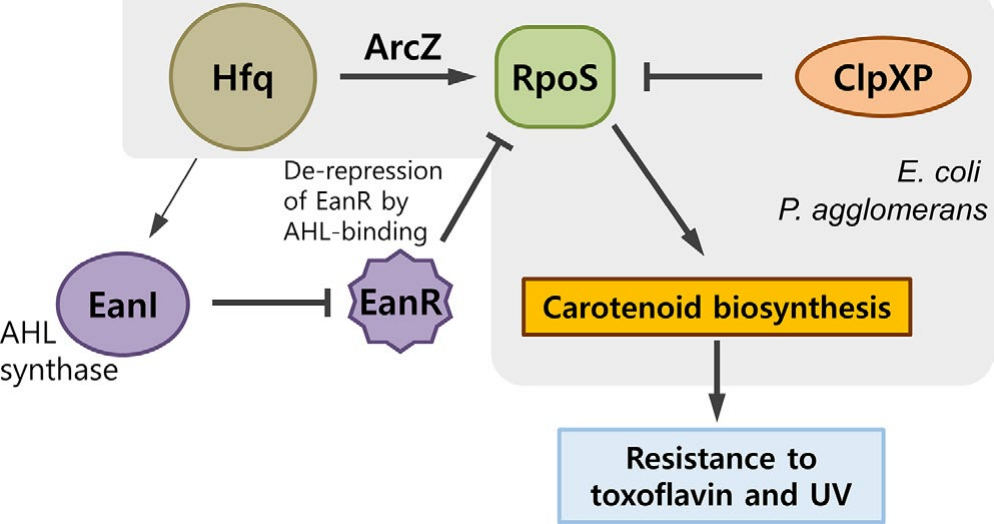
Proposed model of carotenoid production for the previously reported regulatory network Hfq^ArcZ^ → RpoS Ͱ ClpXP and that identified here, in which Hfq‐controlled quorum signaling derepresses EanR to activate RpoS expression, thereby initiating carotenoid production. Carotenoid production confers tolerance to toxoflavin and UV radiation

We found that RpoS regulates carotenoid biosynthesis under the control of Hfq, QS, and ClpP. The regulatory networks of Hfq^ArcZ^ →RpoS Ͱ ClpXP for carotenoid production are similar to those of *E. coli*. Here, we elucidated a regulatory network of carotenoid production involving Hfq‐dependent QS‒RpoS in *P. ananatis*. Hfq regulates the full production of QS signals, thereby de‐repressing the EanR negative regulator to initiate RpoS expression (Figure 8).

## 5 CONCLUSIONS

Microbial biotechnology allows bacterial carotenoids to be used as alternatives to plant‐based carotenoids because of the ease of genetic manipulation of prokaryotes compared with eukaryotes, such as yeasts, fungi, and plants. Here, we used SOE by PCR for gene reassembly to redirect carotenoid synthesis from the plant‐pathogenic bacterium *Pantoea ananatis*. Using SOE by PCR, we reassembled *crtE*–*B*, *crtE*–*B*–*I*, and *crtE*–*B*–*I*–*Y* for phytoene, lycopene, and β‐carotene production, respectively, using *E. coli* to express the reassembled operons. We found that carotenoids confer tolerance to the phytotoxin toxoflavin. The carotenoid production of *P. ananatis* depends on RpoS, which is positively regulated by Hfq/ArcZ and negatively by ClpP, similar to an important regulatory network of *E. coli*, Hfq^ArcZ^ → RpoS Ͱ ClpXP. We also demonstrated that carotenoid production is regulated by Hfq‐controlled QS since the EanR negative regulator on RpoS must be expressed in the stationary phase.

## CONFLICT OF INTEREST

None declared.

## AUTHOR CONTRIBUTIONS

**Okhee Choi:** Conceptualization (lead); Data curation (lead); Formal analysis (lead); Writing‐original draft (lead). **Byeongsam Kang:** Data curation (equal); Methodology (equal); Writing‐original draft (supporting); Writing‐review & editing (supporting). **Yongsang Lee:** Data curation (supporting); Formal analysis (supporting); Methodology (supporting); Writing‐original draft (supporting). **Yeyeong Lee:** Data curation (supporting); Formal analysis (supporting); Writing‐original draft (supporting). **Jinwoo Kim:** Conceptualization (lead); Data curation (lead); Formal analysis (lead); Funding acquisition (lead); Investigation (lead); Methodology (lead); Supervision (lead); Validation (equal); Visualization (lead); Writing‐original draft (lead); Writing‐review & editing (lead).

## ETHICS STATEMENT

None required.

## Data Availability

All data generated or analyzed during this study are included in this paper.

## Appendix

**TABLE A1:** Bacterial strains and plasmids used in this study

Strain or plasmid	Relevant features^a^	Reference
*Escherichia coli*		
DH5α	Cloning host	Bibco BRL
S17‐1 *λpir*	IncP conjugal donor	Promega
*Pantoea ananatis*		
PA13	Wild‐type, Rif^R^	Choi, Kim, et al. (2012))
PA13L	PA13 *lacZY* non‐polar deletion	This study
∆*hfq*	PA13 *hfq* non‐polar deletion	This study
∆*eanI*	PA13 *eanI* non‐polar deletion	This study
∆*eanR*	PA13 *eanR* non‐polar deletion	This study
∆*eanIR*	PA13 *eanIR* non‐polar deletion	This study
∆*rpoS*	PA13 *rpoS* non‐polar deletion	This study
∆*arcZ*	PA13 *arcZ* non‐polar deletion	This study
∆*clpP*	PA13 *clpP* non‐polar deletion	This study
*eanI*‒*lacZY*	PA13L *eanI*::pCOK153, *eanI* ^‒^, Km^R^	This study
*eanI*‒*lacZY* ∆*hfq*	PA13L ∆*hfq eanI*::pCOK153, *eanI* ^‒^, *hfq* ^‒^, Km^R^	This study
*rpoS*‒*lacZY*	PA13L *rpoS*::pYS88, *rpoS* ^‒^, Km^R^	This study
*rpoS*‒*lacZY* ∆*hfq*	PA13L ∆*hfq rpoS*::pYS88, *rpoS* ^‒^, *hfq* ^‒^, Km^R^	This study
*rpoS*‒*lacZY* ∆*eanI*	PA13L ∆*eanI rpoS*::pYS88, *rpoS* ^‒^, *eanI* ^‒^, Km^R^	This study
*rpoS*‒*lacZY* ∆*eanR*	PA13L ∆*eanI rpoS*::pYS88, *rpoS* ^‒^, *eanR* ^‒^, Km^R^	This study
*rpoS*‒*lacZY* ∆*eanIR*	PA13L ∆*eanI rpoS*::pYS88, *rpoS* ^‒^, *eanI* ^‒^, *R* ^‒^, Km^R^	This study
*rpoS*‒*lacZY* ∆*arcZ*	PA13L ∆*arcZ rpoS*::pYS88, *rpoS* ^‒^, *arcZ* ^‒^, Km^R^	This study
*rpoS*‒*lacZY* ∆*clpP*	PA13L ∆*clpP rpoS*::pYS88, *rpoS* ^‒^, *clpP* ^‒^, Km^R^	This study
*crtE*‒*lacZY*	PA13L *crtE*::pCOK184, *crtE* ^‒^, Km^R^	This study
*crtE*‒*lacZY* ∆*eanI*	PA13L ∆*eanI crtE*::pCOK184, *crtE* ^‒^, *eanI* ^‒^, Km^R^	This study
*crtE*‒*lacZY* ∆*rpoS*	PA13L ∆*rpoS crtE*::pCOK184, *crtE* ^‒^, *rpoS* ^‒^, Km^R^	This study
*Plasmids*		
pGEM‐T Easy	PCR cloning vector, Amp^R^	Promega
pVIK112	*lacZY* transcriptional fusion, Km^R^	Kalogeraki and Winans (1997)
pNPTS138‐R6KT	*mobRP4*_*ori*‐R6K *sacB*; suicide plasmid for in‐frame deletions, Km^R^	Lassak et al. (2010)
pBBR1MCS5	pBBR1MCS‐5‐derived broad‐host‐range expression vector containing P*lac* and *lacI* ^q^, *lacZ*α^+^, and Gm^R^	Kovach et al. (1995)
pLAFR3	Cosmid cloning vector, Tet^R^	Keen et al. (1988)
pSRKGm	BHR P*_lac_* expression vector, Gm^R^	Khan et al. (2008)
pCOK153	pVIK112 carrying *eanI* internal fragment, transcriptional fusion	This study
pBS28	pLAFR3::*arcZ* region, Tet^R^; *arcZ* complementation	This study
pCOK125	pNPTS138‐R6KT::*eanR* deletion fragment, Km^R^	This study
pCOK151	pNPTS138‐R6KT::*eanI* deletion fragment, Km^R^	This study
pCOK184	pVIK112 carrying *crtE* internal fragment, transcriptional fusion	This study
pCOK197	pBBR1MCS5::P*_lac_*‒*eanI*; *eanI* complementation	This study
pCOK199	pBBR1MCS5::P*_lac_*‒*eanR*; *eanR* complementation	This study
pCOK218	pBBR1MCS5::*crtEXYIBZ*; Gm^R^	This study
pCOK291	pNPTS138‐R6KT::*rpoS* deletion fragment, Km^R^	This study
pCOK312	pBBR1MCS5::P*_lac_*‒*rpoS*; *rpoS* complementation	This study
pCOK313	pNPTS138‐R6KT::*eanIR* deletion fragment, Km^R^	This study
pCOK318	pNPTS138‐R6KT::*hfq* deletion fragment, Km^R^	This study
pCOK335	pLAFR3::*hfq* region, Tet^R^; *hfq* complementation	This study
pCOK386	pNPTS138‐R6KT::*arcZ* deletion fragment, Km^R^	This study
pOR78	pBBR1MCS5::P*_lac_*‒*clpP*; *clpP* complementation	This study
pYS63	pSRKGm::*rpoS*; *rpoS* complementation	This study
pYS69	pBBR1MCS5::*crtEB*; Gm^R^	This study
pYS71	pBBR1MCS5::*crtEBI*; Gm^R^	This study
pYS76	pBBR1MCS5::*crtEBIYB*; Gm^R^	This study
pYS88	pVIK112 carrying *rpoS* internal fragment, transcriptional fusion	This study

^a^ Amp^R^, ampicillin resistance; Gm^R^, gentamicin resistance; Km^R^, kanamycin resistance; Rif^R^, rifampicin resistance, Tet^R^, tetracycline resistance.

**TABLE A2:** Primers used in SOE by PCR

Primer	Sequences (5’→3’)
a	5’‐GGCctgcagCTGAAACAGGAAACAGCTATGACGGTCTGCGCAAAA‐3’
b	5’‐ATCAGATCCTCCAGCATCAAACCTGCTTTAACTGACGGCAGCGAGTTT‐3’
c	5’‐AGCAGGTTTGATGCTGGAGGATCTGATATGAATAATCCGTCGTTACTC‐3’
d	5’‐GTAGTCGCTCTTTAACGATGAGTCGTCATTCAGAGCGGGCGCTGCCAGAG‐3’
e	5’‐ATGACGACTCATCGTTAAAGAGCGACTACATGAAACCAACTACGGTAATT‐3’
f	5’‐AGCCGCTCCCACTTAAGACAGGCTGACCGTCAAATCAGATCCTCCAGCAT‐3’
g	5’‐CGGTCAGCCTGTCTTAAGTGGGAGCGGCTATGCAACCGCATTATGATCTG‐3’
h	5’‐AATTTCTCCGGTAGAGACGTCTGGCAGCATTAACGATGTGTCGTCATAAT‐3’

Primers ‘b’ and ‘c’, ‘d’ and ‘e’, and ‘f’ and ‘g’ are the SOEing primers in which overlapping sequences are underlined. The *Xho*I restriction site introduced into primer ‘a’ is shown in lowercase.

**TABLE A3:** Primers used in this study

Primers	Description	Uses	References
RT1	5′‐TTCTGCTTCCTGCACCAAAC‐3′	RT‐PCR analysis	This study
RT2	5′‐CTTCCCGGATGCGGGCTCAT‐3′	RT‐PCR analysis	This study
RT3	5′‐GATATCATGGGCCATAGC‐3′	RT‐PCR analysis	This study
RT4	5′‐CAGCAGTTCGACATACTCTT‐3′	RT‐PCR analysis	This study
RT5	5′‐TGAAATGACCACGTATGATT‐3′	RT‐PCR analysis	This study
PCR1f	5′‐ATCTTCATCTTGCCAGTGAG‐3′	RT‐PCR analysis	This study
PCR1r	5′‐TGTTTAATATCGTATTGCTG‐3′	RT‐PCR analysis	This study
PCR2f	5′–CATGGCGAAAATCCAGACCG‐3′	RT‐PCR analysis	This study
PCR2r	5′‐CAGGTTGCTGCTGCTGAAGA‐3′	RT‐PCR analysis	This study
PCR3f	5′‐GATCGGCTACGTATTCTGAG‐3′	RT‐PCR analysis	This study
PCR3r	5′‐CGTTGTTCAAGCAGTAAGAC‐3′	RT‐PCR analysis	This study
PCR4f	5′‐CATTCCTGGCGTCATCGGCT‐3′	RT‐PCR analysis	This study
PCR4r	5′‐AATAACATCGTCACAATGGC‐3′	RT‐PCR analysis	This study
PCR5f	5′‐TGCGCATCTCTGGCAGCGCC‐3′	RT‐PCR analysis	This study
PCR5r	5′‐TTCCGCTATATTCCACGCAA‐3′	RT‐PCR analysis	This study
PCR6f	5′‐ATCTTCATCTTGCCAGTGAG‐3′	RT‐PCR analysis	This study
PCR6r	5′‐AATAACATCGTCACAATGGC‐3′	RT‐PCR analysis	This study
PCR7f	5′‐TGCGCATCTCTGGCAGCGCC‐3′	RT‐PCR analysis	This study
PCR7r	5′‐CGTTGTTCAAGCAGTAAGAC‐3′	RT‐PCR analysis	This study
PCR8f	5′‐CATTCCTGGCGTCATCGGCT‐3′	RT‐PCR analysis	This study
PCR8r	5′‐CAGGTTGCTGCTGCTGAAGA‐3′	RT‐PCR analysis	This study
HFQ1	5'‐GGCACTAGTACTGTTGCATCAACGCAT‐3’	*hfq* deletion	This study
HFQ2	5'‐AAGCTTGGTACCGAATTCCATTCTATCTTTTCCTTATATGCT‐3’	*hfq* deletion	This study
HFQ3	5'‐GAATTCGGTACCAAGCTTGCAGAGTAAGGCGGCACCGTTTAA−3’	*hfq* deletion	This study
HFQ4	5'‐GGCGCATGCCAATTTACCTTCGTGGGT‐3’	*hfq* deletion	This study
HFQP	5'‐GGCCTCGAGCTGAAACAGGAAACAGCTATGGCTAAGGGGCAATCATTA‐3’	*hfq* complementation	This study
HFQX	5'‐GGCCTGCAGGCCTTACTCTGCGTTATCACC‐3’	*hfq* complementation	This study
EanI_1	5'‐ACTAGTTCAAGTATTAGTAGTCTG‐3’	*eanI* deletion	This study
EanI_2	5'‐AAGCTTGGTACCGAATTCACTGACGTCAAACAGTTCAAGCAT‐3’	*eanI* deletion	This study
EanI_3	5'‐GAATTCGGTACCAAGCTTGATATTGTTGCACGTACGGGCTGC‐3’	*eanI* deletion	This study
EanI_4	5'‐GCATGCACCATATGAACAACCTGG‐3’	*eanI* deletion	This study
EanIX	5'‐GGCCTCGAGCTGAAACAGGAAACAGCTATGCTTGAACTGTTTGACGTC‐3’	*eanI* complementation	This study
EanIP	5'‐GGCCTGCAGGTGTTGAGTTAGATCTTATCA‐3’	*eanI* complementation	This study
EanI‐1E‐1	5'‐AAACCACACGTTCAGAAGAAC‐3’	*eanI lacZY* insertion	This study
EanI‐2K	5'‐GGTACCACACTAACTGCCCTTCGCAG‐3’	*eanI lacZY* insertion	This study
RpoS_1	5'‐GGCACTAGTAGTACAACTAACAGTTCA‐3’	*rpoS* deletion	This study
RpoS_2	5'‐AAGCTTGGTACCGAATTCCGTATTCTGGCTCATAAGTGGCTC‐3’	*rpoS* deletion	This study
RpoS_3	5'‐GAATTCGGTACCAAGCTTGAATAAGCCGTCACAGAGCACATC‐3’	*rpoS* deletion	This study
RpoS_4	5'‐GGCGCATGCTGTCTGAGAAACCTCACT‐3’	*rpoS* deletion	This study
RpoS5	5'‐GGCCTCGAGCTGAAACAGGAAACAGCTATGAGCCAGAATACGCTGAAA‐3’	*rpoS* complementation	This study
RpoS6	5'‐GGCGGATCCTTATTCGCGGAACAGTGCTTC‐3’	*rpoS* complementation	This study
RpoSE	5'‐GAGGAAGAAGTTCTCTTT‐3’	*rpoS lacZY* insertion	This study
RpoSK	5'‐GGTACCTTGTTCTGCAATTTCTTC‐3’	*rpoS lacZY* insertion	This study
ArcZ_1	5'‐GGCACTAGTAGTGACCTTGCTAAGGGA‐3’	*arcZ* deletion	This study
ArcZ_2	5'‐AAGCTTGGTACCGAATTCAACACCTTAGCAAATTCAAATTAC‐3’	*arcZ* deletion	This study
ArcZ_3	5'‐GAATTCGGTACCAAGCTTGGCTGGGGTCATTTTTTTGTATCA‐3’	*arcZ* deletion	This study
ArcZ_4	5'‐GGCGCATGCTATCAGTCTGAGAGCGAT‐3’	*arcZ* deletion	This study
arcZH	5'‐GGCAAGCTTATGAGATAAGATCCGGTG‐3’	*arcZ* complementation	This study
arcZB	5'‐ GGCGGATCCTTACAGCATCTTCAGCAG‐3’	*arcZ* complementation	This study
CrtEE	5'‐GACGGATTACTGGATTTGGCC‐3’	*crtE lacZY* insertion	This study
CrtEK	5'‐GGTACCCTGCATGGAGGCACAAAACAG‐3’	*crtE lacZY* insertion	This study
EanR_1	5'‐ACTAGTTTTACCTGCCTGGATCGC‐3’	*eanR* deletion	This study
EanR_2	5'‐AAGCTTGGTACCGAATTCCTATCAGACTGGGTGTTGAGTTAG‐3’	*eanR* deletion	This study
EanR_3	5'‐GAATTCGGTACCAAGCTTTATGTAAGTCTGAAGCGTATCCGT‐3’	*eanR* deletion	This study
EanR_4	5'‐GCATGCGCACAGGTGAAGGCTAAC‐3’	*eanR* deletion	This study
EanRE	5'‐TAGTTTGTGAAGTCTGGC‐3’	*eanR lacZY* insertion	This study
EanRK	5'‐GGTACCAATCAGAACATTTGAAGG‐3’	*eanR lacZY* insertion	This study
JWK5	5'‐GGCCTCGAGCTGAAACAGGAAACAGCTATGTTTTCTTTTTTTCTTGAA‐3’	*eanR* complementation	This study
JWK6	5'‐GGCCTGCAGTGGGGATATTGTTGCACGTAC‐3’	*eanR* complementation	This study
clpE	5'‐TTAAAGAACGCGTAATCT‐3’	*clpP lacZY* insertion	This study
clpK	5'‐GGTACCAATCATCACGCGTGAGTT‐3’	*clpP lacZY* insertion	This study
clpP_1	5'‐GGCACTAGTTACAGCAAGAATAACGAG‐3’	*clpP deletion*	This study
clpP_2	5'‐AAGCTTGGTACCGAATTCCATAATTTCGTTGGCAACATTCTG‐3’	*clpP deletion*	This study
clpP_3	5'‐GAATTCGGTACCAAGCTTTAAGCCCTAACTTCGCCTTGTCTC‐3’	*clpP deletion*	This study
clpP_4	5'‐GGCGCATGCACGTTTGTAGTGGTTGTA‐3’	*clpP deletion*	This study
OR3	5'‐GGCGAATTCCTGAAACAGGAAACAGCTATGTCATACAGTGGCGATCGT‐3’	*clpP* complementation	This study
OR4	5'‐GGCGGATCCTTATTGGCGATGCGTCAGGAT‐3’	*clpP* complementation	This study

## References

[mbo31143-bib-0001] Aiba, H. (2007). Mechanism of RNA silencing by Hfq‐binding small RNAs. Current Opinion in Microbiology, 10, 134–139. 17383928 10.1016/j.mib.2007.03.010

[mbo31143-bib-0002] Bak, G. , Han, K. , Kim, D. , & Lee, Y. (2014). Roles of *rpoS*‐activating small RNAs in pathways leading to acid resistance of *Escherichia coli* . Microbiologyopen, 3, 15–28. 24319011 10.1002/mbo3.143 PMC3937726

[mbo31143-bib-0003] Bardill, J. P. , & Hammer, B. K. (2012). Non‐coding sRNAs regulate virulence in the bacterial pathogen *Vibrio cholerae* . RNA Biology, 9, 392–401. 22546941 10.4161/rna.19975 PMC3384565

[mbo31143-bib-0005] Beck von Bodman, S. , & Farrand, S. K. (1995). Capsular polysaccharide biosynthesis and pathogenicity in *Erwinia stewartii* require induction by an N‐acylhomoserine lactone autoinducer. Journal of Bacteriology, 177, 5000–5008. 7665477 10.1128/jb.177.17.5000-5008.1995 PMC177277

[mbo31143-bib-0006] Becker‐Hapaka, M. , Troxtel, E. , Hoerter, J. , & Eisenstark, A. (1997). RpoS dependent overexpression of carotenoids from *Erwinia herbicola* in OXYR defficient *Escherichia coli* . Biochemical and Biophysical Research Communications, 239, 305–309. 9345315 10.1006/bbrc.1997.7469

[mbo31143-bib-0007] Beisel, C. L. , & Storz, G. (2010). Base pairing small RNAs and their roles in global regulatory networks. FEMS Microbiology Reviews, 34, 866–882. 20662934 10.1111/j.1574-6976.2010.00241.x PMC2920360

[mbo31143-bib-0008] Brennan, R. G. , & Link, T. M. (2007). Hfq structure, function and ligand binding. Current Opinion in Microbiology, 10, 125–133. 17395525 10.1016/j.mib.2007.03.015

[mbo31143-bib-0009] Brown, L. , & Elliott, T. (1996). Efficient translation of the RpoS sigma factor in *Salmonella typhimurium* requires host factor I, an RNA‐binding protein encoded by the *hfq* gene. Journal of Bacteriology, 178, 3763–3770. 8682778 10.1128/jb.178.13.3763-3770.1996 PMC232634

[mbo31143-bib-0010] Chao, Y. , & Vogel, J. (2010). The role of Hfq in bacterial pathogens. Current Opinion in Microbiology, 13, 24–33. 20080057 10.1016/j.mib.2010.01.001

[mbo31143-bib-0011] Choi, O. , Bae, J. , Kang, B. , Lee, Y. , Kim, S. , Fuqua, C. , & Kim, J. (2019). Simple and economical biosensors for distinguishing *Agrobacterium*‐mediated plant galls from nematode‐mediated root knots. Scientific Reports, 9, 17961. 31784634 10.1038/s41598-019-54568-2 PMC6884505

[mbo31143-bib-0012] Choi, O. , Kim, H. , Lee, Y. , Kim, J. , Moon, J. , & Hwang, I. (2012). First report of sheath rot of rice caused by *Pantoea ananatis* in Korea. The Plant Pathology Journal, 28, 331.

[mbo31143-bib-0013] Choi, O. , Lim, J. Y. , Seo, Y.‐S. , Hwang, I. , & Kim, J. (2012). Complete genome sequence of the rice pathogen *Pantoea ananatis* strain PA13. Journal of Bacteriology, 194, 531. 22207741 10.1128/JB.06450-11 PMC3256675

[mbo31143-bib-0014] Coutinho, T. A. , & Venter, S. N. (2009). *Pantoea ananatis*: an unconventional plant pathogen. Molecular Plant Pathology, 10, 325–335. 19400836 10.1111/j.1364-3703.2009.00542.x PMC6640510

[mbo31143-bib-0015] De Baere, T. , Verhelst, R. , Labit, C. , Verschraegen, G. , Wauters, G. , Claeys, G. , & Vaneechoutte, M. (2004). Bacteremic infection with *Pantoea ananatis* . Journal of Clinical Microbiology, 42, 4393–4395. 15365053 10.1128/JCM.42.9.4393-4395.2004 PMC516322

[mbo31143-bib-0016] Ding, Y. , Davis, B. M. , & Waldor, M. K. (2004). Hfq is essential for *Vibrio cholera* virulence and downregulates sigma expression. Molecular Microbiology, 53, 345–354. 15225327 10.1111/j.1365-2958.2004.04142.x

[mbo31143-bib-0017] Duan, X. , Chen, J. , & Wu, J. (2013). Improving the thermostability and catalytic efficiency of *Bacillus deramificans* pullulanase by site‐directed mutagenesis. Applied and Environmental Microbiology, 79, 4072–4077. 23624477 10.1128/AEM.00457-13 PMC3697558

[mbo31143-bib-0018] Dufossé, L. , Galaup, P. , Yaron, A. , Arad, S. M. , Blanc, P. , Murthy, K. N. C. , & Ravishankar, G. A. (2005). Microorganisms and microalgae as sources of pigments for food use: a scientific oddity or an industrial reality? Trends in Food Science and Technology, 16, 389–406.

[mbo31143-bib-0019] Dundas, I. D. , & Larsen, H. (1963). A study on the killing by light of photosensitized cells of *Halobacterium salinarium* . Archiv für Mikrobiologie, 46, 19–28. 14054143 10.1007/BF00406383

[mbo31143-bib-0020] Dutta, B. , Gitaitis, R. , Barman, A. , Avci, U. , Marasigan, K. , & Srinivasan, R. (2016). Interactions between *Frankliniella fusca* and *Pantoea ananatis* in the center rot epidemic on onion (*Allium cepa*). Phytopathology, 106, 956–962. 27135678 10.1094/PHYTO-12-15-0340-R

[mbo31143-bib-0021] Fasano, E. , Serini, S. , Mondella, N. , Trombino, S. , Celleno, L. , Lanza, P. , Cittadini, A. , & Calviello, G. (2014). Antioxidant and anti‐Inflammatory effects of selected natural compounds contained in a dietary supplement on two human immortalized keratinocyte lines. BioMed Research International, 2014, 1–11. 10.1155/2014/327452 PMC4150458 25197638

[mbo31143-bib-0022] Fidan, O. , & Zhan, J. (2019). Discovery and engineering of an endophytic *Pseudomonas* strain from *Taxus chinensis* for efficient production of zeaxanthin diglucoside. Journal of Biological Engineering, 13, 66. 31388354 10.1186/s13036-019-0196-x PMC6676639

[mbo31143-bib-0023] Figueroa‐Bossi, N. , Lemire, S. , Maloriol, D. , Balbontín, R. , Casadesús, J. , & Bossi, L. (2006). Loss of Hfq activates the sigmaE‐dependent envelope stress response in *Salmonella enterica* . Molecular Microbiology, 62, 838–852. 16999834 10.1111/j.1365-2958.2006.05413.x

[mbo31143-bib-0024] Fröhlich, K. S. , & Vogel, J. (2009). Activation of gene expression by small RNA. Current Opinion in Microbiology, 12, 674–682. 19880344 10.1016/j.mib.2009.09.009

[mbo31143-bib-0025] Fuqua, C. , Winans, S. C. , & Greenberg, E. P. (1996). Census and consensus in bacterial ecosystems: the LuxR‐LuxI family of quorum‐sensing transcriptional regulators. Annual Review of Microbiology, 50, 727–751. 10.1146/annurev.micro.50.1.727 8905097

[mbo31143-bib-0026] Gaida, S. M. , Al‐Hinai, M. A. , Indurthi, D. C. , Nicolaou, S. A. , & Papoutsakis, E. T. (2013). Synthetic tolerance: three noncoding small RNAs, DsrA, ArcZ and RprA, acting supra‐additively against acid stress. Nucleic Acids Research, 41, 8726–8737. 23892399 10.1093/nar/gkt651 PMC3794604

[mbo31143-bib-0027] Gao, M. , Tang, M. , Guerich, L. , Salas‐Gonzalez, I. , & Teplitski, M. (2015). Modulation of *Sinorhizobium meliloti* quorum sensing by Hfq‐mediated post‐transcriptional regulation of ExpR. Environmental Microbiology Reports, 7, 148–154. 25382642 10.1111/1758-2229.12235

[mbo31143-bib-0028] Gish, W. , & States, D. J. (1993). Identification of protein coding regions by database similarity search. Nature Genetics, 3, 266–272. 8485583 10.1038/ng0393-266

[mbo31143-bib-0029] Gottesman, S. , McCullen, C. A. , Guillier, M. , Vanderpool, C. K. , Majdalani, N. , Benhammou, J. , Thompson, K. M. , FitzGerald, P. C. , Sowa, N. A. , & FitzGerald, D. J. (2006). Small RNA regulators and the bacterial response to stress. Cold Spring Harbor Symposia on Quantitative Biology, 71, 1–11. 17381274 10.1101/sqb.2006.71.016 PMC3592358

[mbo31143-bib-0030] Guerinot, M. L. (2000). The green revolution strikes gold. Science, 287, 241–243. 10660423 10.1126/science.287.5451.241

[mbo31143-bib-0031] Higuchi, R. , Krummel, B. , & Saiki, R. K. (1988). A general method of *in vitro* preparation and specific mutagenesis of DNA fragments: study of protein and DNA interactions. Nucleic Acids Research, 15, 7351–7367. 10.1093/nar/16.15.7351 PMC338413 3045756

[mbo31143-bib-0032] Ho, S. N. , Hunt, H. D. , Horton, R. M. , Pullen, J. K. , & Pease, L. R. (1989). Site‐directed mutagenesis by overlap extension using the polymerase chain reaction. Gene, 77, 51–59. 2744487 10.1016/0378-1119(89)90358-2

[mbo31143-bib-0033] Horton, R. M. , Cai, Z. , Ho, S. N. , & Pease, L. R. (1995). Gene splicing by overlap extension: tailor‐made genes using the polymerase chain reaction. BioTechniques, 54, 528–535. 2357375

[mbo31143-bib-0034] Horton, R. M. , Hunt, H. D. , Ho, S. N. , Pullen, J. K. , & Pease, L. R. (1989). Engineering hybrid genes without the use of restriction enzymes: gene splicing by overlap extension. Gene, 77, 61–68. 2744488 10.1016/0378-1119(89)90359-4

[mbo31143-bib-0035] Hundle, B. , Alberti, M. , Nievelstein, V. , Beyer, P. , Kleinig, H. , Armstrong, G. A. , Burke, D. H. , & Hearst, J. E. (1994). Functional assignment of *Erwinia herbicola* Eho10 carotenoid genes expressed in *Escherichia coli* . Molecular and General Genetics, 245, 406–416. 7808389 10.1007/BF00302252

[mbo31143-bib-0036] Hussain, H. , & Chong, N. (2016). Combined overlap extension PCR method for improved site directed mutagenesis. BioMed Research International, 2016, 8041532. 27995143 10.1155/2016/8041532 PMC5138438

[mbo31143-bib-0037] Hwang, W. , Arluison, V. , & Hohng, S. (2011). Dynamic competition of DsrA and *rpoS* fragments for the proximal binding site of Hfq as a means for efficient annealing. Nucleic Acids Research, 39, 5131–5139. 21357187 10.1093/nar/gkr075 PMC3130260

[mbo31143-bib-0038] Kalogeraki, V. S. , & Winans, S. C. (1997). Suicide plasmids containing promoterless reporter genes can simultaneously disrupt and create fusions to target genes of diverse bacteria. Gene, 188, 69–75. 9099861 10.1016/s0378-1119(96)00778-0

[mbo31143-bib-0039] Kang, B. (2017). Hfq regulates Pathogenicity in Pantoea ananatis. Master of Science Thesis, Gyeongsang National University.

[mbo31143-bib-0040] Kay, E. , Humair, B. , Dénervaud, V. , Riedel, K. , Spahr, S. , Eberl, L. , Valverde, C. , & Haas, D. (2006). Two GacA‐dependent small RNAs modulate the quorum‐sensing response in *Pseudomonas aeruginosa* . Journal of Bacteriology, 188, 6026–6033. 16885472 10.1128/JB.00409-06 PMC1540078

[mbo31143-bib-0041] Keen, N. T. , Tamaki, S. , Kobayashi, D. , & Trollinger, D. (1988). Improved broad‐host‐range plasmids for DNA cloning in gram‐negative bacteria. Gene, 70, 191–197. 2853689 10.1016/0378-1119(88)90117-5

[mbo31143-bib-0042] Khan, S. R. , Gaines, J. , Roop, R. M. 2nd , & Farrand, S. K. (2008). Broad‐host‐range expression vectors with tightly regulated promoters and their use to examine the influence of TraR and TraM expression on Ti plasmid quorum sensing. Applied and Environmental Microbiology, 74, 5053–5062. 18606801 10.1128/AEM.01098-08 PMC2519271

[mbo31143-bib-0043] Kim, J. , & Choi, O. (2012). An outbreak of onion center rot caused by *Pantoea ananatis* in Korea. Plant Disease, 96, 1576. 10.1094/PDIS-03-12-0251-PDN 30727345

[mbo31143-bib-0044] Kim, J. , Heindl, J. E. , & Fuqua, C. (2013). Coordination of division and development influences complex multicellular behavior in *Agrobacterium tumefaciens* . PLoS One, 8, e56682. 23437210 10.1371/journal.pone.0056682 PMC3577659

[mbo31143-bib-0045] Kim, J. , Kim, J.‐G. , Kang, Y. , Jang, J. Y. , Jog, G. J. , Lim, J. Y. , Kim, S. , Suga, H. , Nagamatsu, T. , & Hwang, I. (2004). Quorum sensing and the LysR‐type transcriptional activator ToxR regulate toxoflavin biosynthesis and transport in *Burkholderia glumae* . Molecular Microbiology, 54, 921–934. 15522077 10.1111/j.1365-2958.2004.04338.x

[mbo31143-bib-0046] Kirti, K. , Amita, S. , Priti, S. , Kumar, A. M. , & Jyoti, S. (2014). Colorful world of microbes: Carotenoids and their applications. Advanced Biology, 2014, 13.

[mbo31143-bib-0047] Kovach, M. E. , Elzer, P. H. , Hill, D. S. , Robertson, G. T. , Farris, M. A. , Roop, R. M. , & Peterson, K. M. (1995). Four new derivatives of the broad‐host‐range cloning vector pBBR1MCS, carrying different antibiotic‐resistance cassettes. Gene, 166, 175–176. 8529885 10.1016/0378-1119(95)00584-1

[mbo31143-bib-0048] Kunisawa, R. , & Stanier, R. Y. (1958). Studies on the role of carotenoids pigments in a chemoheterotrophic bacterium, *Corynebacterium poinsettiae* . Archiv für Mikrobiologie, 31, 146–156.

[mbo31143-bib-0049] Lassak, J. , Henche, A. L. , Binnenkade, L. , & Thormann, K. M. (2010). ArcS, the cognate sensor kinase in an atypical Arc system of *Shewanella oneidensis* MR‐1. Applied and Environmental Microbiology, 76, 3263–3274. 20348304 10.1128/AEM.00512-10 PMC2869118

[mbo31143-bib-0050] Latuasan, H. E. , & Berends, W. (1961). On the origin of the toxicity of toxoflavin. Biochimica et Biophysica Acta, 52, 502–508. 14462713 10.1016/0006-3002(61)90408-5

[mbo31143-bib-0051] Lee, P. C. , Momen, A. Z. , Mijts, B. N. , & Schmidt‐Dannert, C. (2003). Biosynthesis of structurally novel carotenoids in *Escherichia coli* . Chemical Biology, 10, 453–462. 10.1016/s1074-5521(03)00103-0 12770827

[mbo31143-bib-0052] Lee, Y. S. (2015). Quorum sensing and RpoS regulate carotenoid biosynthesis of Pantoea ananatis PA13. Master of Science Thesis, Gyeongsang National University.

[mbo31143-bib-0053] Lenz, D. H. , Mok, K. C. , Lilley, B. N. , Kulkarni, R. V. , Wingreen, N. S. , & Bassler, B. L. (2004). The small RNA chaperone Hfq and multiple small RNAs control quorum sensing in *Vibrio harveyi* and *Vibrio cholerae* . Cell, 118, 69–82. 15242645 10.1016/j.cell.2004.06.009

[mbo31143-bib-0054] Lorenz, C. , Gesell, T. , Zimmermann, B. , Schoeberl, U. , Bilusic, I. , Rajkowitsch, L. , Waldsich, C. , von Haeseler, A. , & Schroeder, R. (2010). Genomic SELEX for Hfq‐binding RNAs identifies genomic aptamers predominantly in antisense transcripts. Nucleic Acids Research, 38, 3794–3808. 20348540 10.1093/nar/gkq032 PMC2887942

[mbo31143-bib-0055] Lorquin, J. , Molouba, F. , & Dreyfus, B. L. (1997). Identification of the carotenoid pigment canthaxanthin from photosynthetic *Bradyrhizobium* strains. Applied and Environmental Microbiology, 63, 1151–1154. 16535544 10.1128/aem.63.3.1151-1154.1997 PMC1389138

[mbo31143-bib-0056] Lu, Q. , Bu, Y.‐F. , & Liu, J.‐Z. (2017). Metabolic engineering of *Escherichia coli* for producing astaxanthin as the predominant carotenoid. Marine Drugs, 15, 296. 10.3390/md15100296 PMC5666404 28937591

[mbo31143-bib-0057] Lutnaes, B. F. , Strand, Å. , Pétursdóttir, S. K. , & Liaaen‐Jensen, S. (2004). Carotenoids of thermophilic bacteria–*Rhodothermus marinus* from submarine Icelandic hot springs. Biochemical Systematics and Ecology, 32, 455–468.

[mbo31143-bib-0058] Majdalani, N. , Vanderpool, C. K. , & Gottesman, S. (2005). Bacterial small RNA regulators. Critical Reviews in Biochemistry and Molecular Biology, 40, 93–113. 15814430 10.1080/10409230590918702

[mbo31143-bib-0059] Mandin, P. , & Gottesman, S. (2010). Integrating anaerobic/aerobic sensing and the general stress response through the ArcZ small RNA. The EMBO Journal, 29, 3094–3107. 20683441 10.1038/emboj.2010.179 PMC2944060

[mbo31143-bib-0060] Mathews, M. M. , & Sistrom, W. R. (1959). Function of carotenoid pigments in non‐photosynthetic bacteria. Nature, 184, 1892–1896. 14422277 10.1038/1841892a0

[mbo31143-bib-0061] Mathews, M. M. , & Sistrom, W. R. (1960). The function of carotenoid pigments of *Sarcina lutea* . Archiv für Mikrobiologie, 35, 139–146. 14422279 10.1007/BF00425002

[mbo31143-bib-0062] Merritt, P. M. , Danhorn, T. , & Fuqua, C. (2007). Motility and chemotaxis in *Agrobacterium tumefaciens* surface attachment and biofilm formation. Journal of Bacteriology, 189, 8005–8014. 17766409 10.1128/JB.00566-07 PMC2168663

[mbo31143-bib-0063] Mijts, B. N. , & Schmidt‐Dannert, C. (2003). Engineering of secondary metabolite pathways. Current Opinion in Biotechnology, 14, 597–602. 14662388 10.1016/j.copbio.2003.09.009

[mbo31143-bib-0064] Minogue, T. D. , Carlier, A. L. , Koutsoudis, M. D. , & Beck von Bodman, S. (2005). The cell density‐dependent expression of stewartan exopolysaccharide in *Pantoea stewartii* ssp. *stewartii* is a function of EsaR‐mediated repression of the *rcsA* gene. Molecular Microbiology, 56, 189–203. 15773989 10.1111/j.1365-2958.2004.04529.x

[mbo31143-bib-0065] Misawa, N. , Satomi, Y. , Kondo, K. , Yokoyama, A. , Kajiwara, S. , Saito, T. , Ohtani, T. , & Miki, W. (1995). Structure and functional analysis of a marine bacterial carotenoid biosynthesis gene cluster and astaxanthin biosynthetic pathway proposed at the gene level. Journal of Bacteriology, 177, 6575–6584. 7592436 10.1128/jb.177.22.6575-6584.1995 PMC177511

[mbo31143-bib-0066] Mohammadi, M. , Burbank, L. , & Roper, C. (2012). The biological role of pigment production for the bacterial phytopathogen, *Pantoea stewartii* subsp. *stewartii* . Applied and Environmental Microbiology, 78, 6859–6865. 22820327 10.1128/AEM.01574-12 PMC3457488

[mbo31143-bib-0067] Moran, N. A. , & Jarvik, T. (2010). Lateral transfer of genes from fungi underlies carotenoid production in aphids. Science, 328, 624–627. 20431015 10.1126/science.1187113

[mbo31143-bib-0068] Morohoshi, T. , Nakamura, Y. , Yamazaki, G. , Ishida, A. , Kato, N. , & Ikeda, T. (2007). The plant pathogen *Pantoea ananatis* produces *N*‐acylhomoserine lactone and causes center rot disease of onion by quorum sensing. Journal of Bacteriology, 189, 8333–8338. 17827290 10.1128/JB.01054-07 PMC2168703

[mbo31143-bib-0069] Mostofian, B. , Johnson, Q. R. , Smith, J. C. , & Cheng, X. (2020). Carotenoids promote lateral packing and condensation of lipid membranes. Physical Chemistry Chemical Physics, 7, 12281–12293. 10.1039/d0cp01031f 32432296

[mbo31143-bib-0070] Muffler, A. , Fischer, D. , & Hengge‐Aronis, R. (1996). The RNA‐binding protein HF‐I, known as a host factor for phage Qβ RNA replication, is essential‐for *rpoS* translation in *Escherichia coli* . Genes & Development, 10, 1143–1151. 8654929 10.1101/gad.10.9.1143

[mbo31143-bib-0071] Mullis, K. , Faloona, F. , Scharf, S. , Saiki, R. , Horn, G. , & Erlich, H. (1986). Specific enzymatic amplification of DNA *in vitro*: the polymerase chain reaction. Cold Spring Harbor Symposia on Quantitative Biology, L1, 263–273. 10.1101/sqb.1986.051.01.032 3472723

[mbo31143-bib-0072] Nagamatsu, T. , Hashiguchi, Y. , Sakuma, Y. , & Yoneda, F. (1982). Autorecycling oxidation of amines to carbonyl compound catalized by 3, 4‐disubstituted 4‐deazatoxoflavin derivatives. Chemistry Letters, 11, 1309–1312.

[mbo31143-bib-0073] Oren, A. (2009). Microbial diversity and microbial abundance in salt‐saturated brines: Why are the waters of hypersaline lakes red? Natural Resources and Environmental Issues, 15, 49.

[mbo31143-bib-0074] Paliwal, C. , Mitra, M. , Bhayani, K. , Bharadwaj, S. V. , Ghosh, T. , Dubey, S. , & Mishra, S. (2017). Abiotic stresses as tools for metabolites in microalgae. Bioresource Technology, 244, 1216–1226. 28552566 10.1016/j.biortech.2017.05.058

[mbo31143-bib-0075] Papenfort, K. , Said, N. , Welsink, T. , Lucchini, S. , Hinton, J. C. D. , & Vogel, J. (2009). Specific and pleiotropic patterns of mRNA regulation by ArcZ, a conserved, Hfq‐dependent small RNA. Molecular Microbiology, 74, 139–158. 19732340 10.1111/j.1365-2958.2009.06857.x

[mbo31143-bib-0076] Platt, T. G. , & Fuqua, C. (2010). What's in a name? The semantics of quorum sensing. Trends in Microbiology, 18, 383–387. 20573513 10.1016/j.tim.2010.05.003 PMC2932771

[mbo31143-bib-0077] Raju, R. , Goldberg, A. , & Rubin, E. (2012). Bacterial proteolytic complexes as therapeutic targets. Nature Reviews Drug Discovery, 11, 777–789. 23023677 10.1038/nrd3846

[mbo31143-bib-0078] Ram, S. , Mitra, M. , Shah, F. , Tirkey, S. R. , & Mishra, S. (2020). Bacteria as an alternate biofactory for carotenoid production: A review of its applications, opportunities and challenges. Journal of Functional Foods, 67, 103867.

[mbo31143-bib-0079] Ram, S. , Paliwal, C. , & Mishra, S. (2019). Growth medium and nitrogen stress sparked biochemical and carotenogenic alterations in *Scenedesmus* sp. CCNM 1028. Bioresource Technology Reports, 7, 100194.

[mbo31143-bib-0080] Repoila, F. , Majdalani, N. , & Gottesman, S. (2003). Small non‐coding RNAs, co‐ordinators of adaptation processes in *Escherichia coli*: The RpoS paradigm. Molecular Microbiology, 48, 855–861. 12753181 10.1046/j.1365-2958.2003.03454.x

[mbo31143-bib-0081] Sajilata, M. , Singhal, R. , & Kamat, M. (2008). The carotenoid pigment zeaxanthin – A review. Comprehensive Reviews in Food Science and Food Safety, 7, 29–49.

[mbo31143-bib-0082] Sambrook, J. , & Russell, D. W. (2001). Molecular cloning: A laboratory manual. Cold Spring Harbor Laboratory Press.

[mbo31143-bib-0085] Schachterle, J. K. , & Sundin, G. W. (2019). The leucine‐responsive regulatory protein participates in virulence regulation downstream of small RNA ArcZ in Erwinia amylovora. MBio, 10, e00757‐19. 31138749 10.1128/mBio.00757-19 PMC6538786

[mbo31143-bib-0086] Sedkova, N. , Tao, L. , Rouvière, P. E. , & Cheng, Q. (2005). Diversity of carotenoid synthesis gene clusters from environmental *Enterobacteriaceae* strains. Applied and Environmental Microbiology, 71, 8141–8146. 16332796 10.1128/AEM.71.12.8141-8146.2005 PMC1317436

[mbo31143-bib-0087] Shin, G. Y. , Schachterle, J. K. , Shyntum, D. Y. , Moleleki, L. N. , Coutinho, T. A. , & Sundin, G. W. (2019). Functional characterization of a global virulence regulator Hfq and identification of Hfq‐dependent sRNAs in the plant pathogen *Pantoea ananatis* . Frontiers in Microbiology, 10, 2075. 31572315 10.3389/fmicb.2019.02075 PMC6749038

[mbo31143-bib-0088] Sittka, A. , Pfeiffer, V. , Tedin, K. , & Vogel, J. (2007). The RNA chaperone Hfq is essential for the virulence of *Salmonella typhimurium* . Molecular Microbiology, 63, 193–217. 17163975 10.1111/j.1365-2958.2006.05489.x PMC1810395

[mbo31143-bib-0089] Song, G. H. , Kim, S. H. , Choi, B. H. , Han, S. J. , & Lee, P. C. (2013). Heterologous carotenoid‐biosynthetic enzymes: functional complementation and effects on carotenoid profiles in *Escherichia coli* . Applied and Environmental Microbiology, 79, 610–618. 23144136 10.1128/AEM.02556-12 PMC3553770

[mbo31143-bib-0090] Soper, T. , Mandin, P. , Majdalani, N. , Gottesman, S. , & Woodson, S. A. (2010). Positive regulation by small RNAs and the role of Hfq. Proceedings of the National Academy of Sciences of the United States of America, 107, 9602–9607. 20457943 10.1073/pnas.1004435107 PMC2906882

[mbo31143-bib-0091] Stanier, R. Y. (1959). Formation and function of the photosynthetic pigment system in purple bacteria. The photochemical apparatus: Its structure and function. Brookhaven Symposium in Biology, 11, 43–53.

[mbo31143-bib-0092] Storz, G. , Altuvia, S. , & Wassarman, K. M. (2005). An abundance of RNA regulators. Annual Review of Biochemistry, 74, 199–217. 10.1146/annurev.biochem.74.082803.133136 15952886

[mbo31143-bib-0093] Storz, G. , Opdyke, A. J. , & Zhang, A. (2004). Controlling mRNA stability and translation with small, noncoding RNAs. Current Opinion in Microbiology, 7, 140–144. 15063850 10.1016/j.mib.2004.02.015

[mbo31143-bib-0094] Tian, B. , & Hua, Y. (2010). Carotenoid biosynthesis in extremophilic *Deinococcus‐Thermus* bacteria. Trends in Microbiology, 18, 512–520. 20832321 10.1016/j.tim.2010.07.007

[mbo31143-bib-0095] Vila, E. , Hornero‐Méndez, D. , Azziz, G. , Lareo, C. , & Saravia, V. (2019). Carotenoids from heterotrophic bacteria isolated from Fildes Peninsula, King George Island, Antarctica. Biotechnology Reports, 21, e00306. 30705834 10.1016/j.btre.2019.e00306 PMC6348148

[mbo31143-bib-0096] Virtamo, J. , Taylor, P. R. , Kontto, J. , Männistö, S. , Utriainen, M. , Weinstein, S. J. , & Albanes, D. (2014). Effects of α‐tocopherol and β‐carotene supplementation on cancer incidence and mortality: 18‐year post‐intervention follow‐up of the alpha‐tocopherol, beta‐carotene cancer prevention (ATBC) study. International Journal of Cancer, 135, 178–185. 24338499 10.1002/ijc.28641 PMC3991754

[mbo31143-bib-0097] Vogel, J. , & Wagner, E. G. H. (2007). Target identification of small noncoding RNAs in bacteria. Current Opinion in Microbiology, 10, 262–270. 17574901 10.1016/j.mib.2007.06.001

[mbo31143-bib-0098] Wang, C. , Pu, T. , Lou, W. , Wang, Y. , Gao, Z. , Hu, B. , & Fan, J. (2018). Hfq, a RNA chaperone, contributes to virulence by regulating plant cell wall–degrading enzyme production, type VI secretion system expression, bacterial competition, and suppressing host defense response in *Pectobacterium carotovorum* . Molecular Plant‐Microbe Interactions, 31, 1166–1178. 30198820 10.1094/MPMI-12-17-0303-R

[mbo31143-bib-0099] Warrens, A. N. , Jones, M. D. , & Lechler, R. I. (1997). Splicing by overlap extension by PCR using asymmetric amplification: an improved technique for the generation of hybrid proteins of immunological interest. Gene, 186, 29–35. 9047341 10.1016/s0378-1119(96)00674-9

[mbo31143-bib-0100] Waters, C. M. , & Bassler, B. L. (2005). Quorum sensing: cell‐to‐cell communication in bacteria. Annual Review of Cell and Developmental Biology, 21, 319–346. 10.1146/annurev.cellbio.21.012704.131001 16212498

[mbo31143-bib-0101] Waters, L. S. , & Storz, G. (2009). Regulatory RNAs in bacteria. Cell, 136, 615–628. 19239884 10.1016/j.cell.2009.01.043 PMC3132550

[mbo31143-bib-0102] Weller‐Stuart, T. , De Maayer, P. , & Coutinho, T. (2017). *Pantoea ananatis*: genomic insights into a versatile pathogen. Molecular Plant Pathology, 18, 1191–1198. 27880983 10.1111/mpp.12517 PMC6638271

[mbo31143-bib-0103] Xiao, Y.‐H. , Yin, M.‐H. , Hou, L. , Luo, M. , & Pei, Y. (2007). Asymmetric overlap extension PCR method bypassing intermediate purification and the amplification of wild‐type template in site‐directed mutagenesis. Biotechnology Letters, 29, 925–930. 17356793 10.1007/s10529-007-9327-4

[mbo31143-bib-0104] Xu, J. , Kim, J. , Benjamin, J. , Koestler, B. J. , Choi, J.‐H. , Waters, C. M. , & Fuqua, C. (2013). Genetic analysis of *Agrobacterium tumefaciens* unipolar polysaccharide production reveals complex integrated control of the motile‐to‐sessile switch. Molecular Microbiology, 89, 929–948. 23829710 10.1111/mmi.12321 PMC3799846

[mbo31143-bib-0105] Zeng, F. , Zhang, Y. , Zhang, Z. , Malik, A. A. , & Lin, Y. (2017). Multiple‐site fragment deletion, insertion and substitution mutagenesis by modified overlap extension PCR. Biotechnology & Biotechnological Equipment, 31, 339–348.

[mbo31143-bib-0106] Zeng, Q. , McNally, R. R. , & Sundin, G. W. (2013). Global small RNA chaperone Hfq and regulatory small RNAs are important virulence regulators in *Erwinia amylovora* . Journal of Bacteriology, 195, 1706–1717. 23378513 10.1128/JB.02056-12 PMC3624556

[mbo31143-bib-0107] Zeng, Q. , & Sundin, G. W. (2014). Genome‐wide identification of Hfq‐regulated small RNAs in the fire blight pathogen *Erwinia amylovora* discovered small RNAs with virulence regulatory function. BMC Genomics, 15, 414. 24885615 10.1186/1471-2164-15-414 PMC4070566

